# Non-Invasive Self-Adaptive Information States’ Acquisition inside Dynamic Scattering Spaces

**DOI:** 10.34133/research.0375

**Published:** 2024-05-31

**Authors:** Ruifeng Li, Jinyan Ma, Da Li, Yunlong Wu, Chao Qian, Ling Zhang, Hongsheng Chen, Tsampikos Kottos, Er-Ping Li

**Affiliations:** ^1^ Zhejiang University–University of Illinois at Urbana-Champaign Institute, Zhejiang University, Haining 314400, China.; ^2^ College of Information Science and Electronic Engineering, Zhejiang University, Hangzhou 310027, China.; ^3^ Wave Transport in Complex Systems Lab, Department of Physics, Wesleyan University, Middletown, CT 06459, USA.

## Abstract

Pushing the information states’ acquisition efficiency has been a long-held goal to reach the measurement precision limit inside scattering spaces. Recent studies have indicated that maximal information states can be attained through engineered modes; however, partial intrusion is generally required. While non-invasive designs have been substantially explored across diverse physical scenarios, the non-invasive acquisition of information states inside dynamic scattering spaces remains challenging due to the intractable non-unique mapping problem, particularly in the context of multi-target scenarios. Here, we establish the feasibility of non-invasive information states’ acquisition experimentally for the first time by introducing a tandem-generated adversarial network framework inside dynamic scattering spaces. To illustrate the framework’s efficacy, we demonstrate that efficient information states’ acquisition for multi-target scenarios can achieve the Fisher information limit solely through the utilization of the external scattering matrix of the system. Our work provides insightful perspectives for precise measurements inside dynamic complex systems.

## Introduction

The information states describe wave field routes used for parameters’ precise estimation or measurement. Enhancing the information states' acquisition efficiency is a long-standing goal, which is unfortunately constrained by system noise [1,2]; in particular, the noise fluctuations inside scattering spaces significantly affect the information state quality. Recently, the emerging electromagnetic information theory [1,3,4] provides a new perspective for describing the information states based on the wave field routes. By elucidating the basic relationship between information states and field distributions with the help of wavefront shaping technology [5,6] and superstructure surface [7,8], the electromagnetic information theory offers profound insights into quantum communication [9], cell manipulation [10], deep tissue imaging [11], wireless charging [12], and other fields [13,14].

To acquire the information states that are crucial for parameters’ precise estimation and even wireless interconnection, a great challenge lies in the random scattering caused by the disorder of complex systems, which leads to signal attenuation and weakens the observable wave field features [15–17]. Endeavors aimed at mitigating random scattering, such as metasurface-enabled wireless communications [18], offer a viable technical approach for controlling wave field. Over the years, numerous operators or matrices based on physical features of wave field properties have been derived [19–28], which provide valuable mathematical tools for investigating the interaction mechanism between the wave field and scattering spaces. However, the dependence on repeated measurements and partially intrusive detection constrains the information states’ acquisition efficiency.

Despite the widespread interest in information states, the implementation of non-invasive design within dynamic scattering spaces poses a formidable challenge due to the seemingly uncontrollable complexity. Although machine-learning-enabled adaptive optics [29–32] and neural network calculating the scattering matrix [33] have found wide application in diverse physical scenarios [34–36], none of these approaches offer a framework for non-invasive self-adaptive information states’ acquisition inside dynamic scattering spaces, primarily due to the non-unique mapping problem. Resolving this challenge would invigorate wavefront shaping [37–40] and specific mode engineering [41–43], liberating existing information states’ operators from the predefined environment, especially for the multi-target scenarios.

In this article, we introduce the concept of “neural route” (Neuroute) and present the non-invasive information states’ acquisition using the Neuroute in our proof-of-principle experiments, demonstrating its feasibility for the first time. To address the non-unique mapping problem, a tandem-generated adversarial network framework is embedded for information states’ acquisition (istGAN) in the Neuroute, aiming to overcome the limitations of prior information states’ operators [23,44,45], which relied on intrusive perturbations to the target or involved time-consuming iterations. Moreover, we investigate 2 fundamental properties of the Neuroute. Firstly, we demonstrate its convergence in a given non-invasive environment, and secondly, we establish its self-adaptability inside dynamic scattering spaces. Additionally, the Neuroute is the first tool to achieve the Fisher information limit in the presence of artificial intelligence, building neural statistic dynamics including generation, classification, and deriving the limit efficiently. From a broader perspective, the architecture of the Neuroute holds promise in facilitating the parameters’ precise estimation and could be applied to coherent measurement scenarios and diverse research domains, addressing challenges related to the manipulation of wave fields.

## Results

### Neuroute enabled non-invasive coherent measurement scenarios

**Fig. 1.**
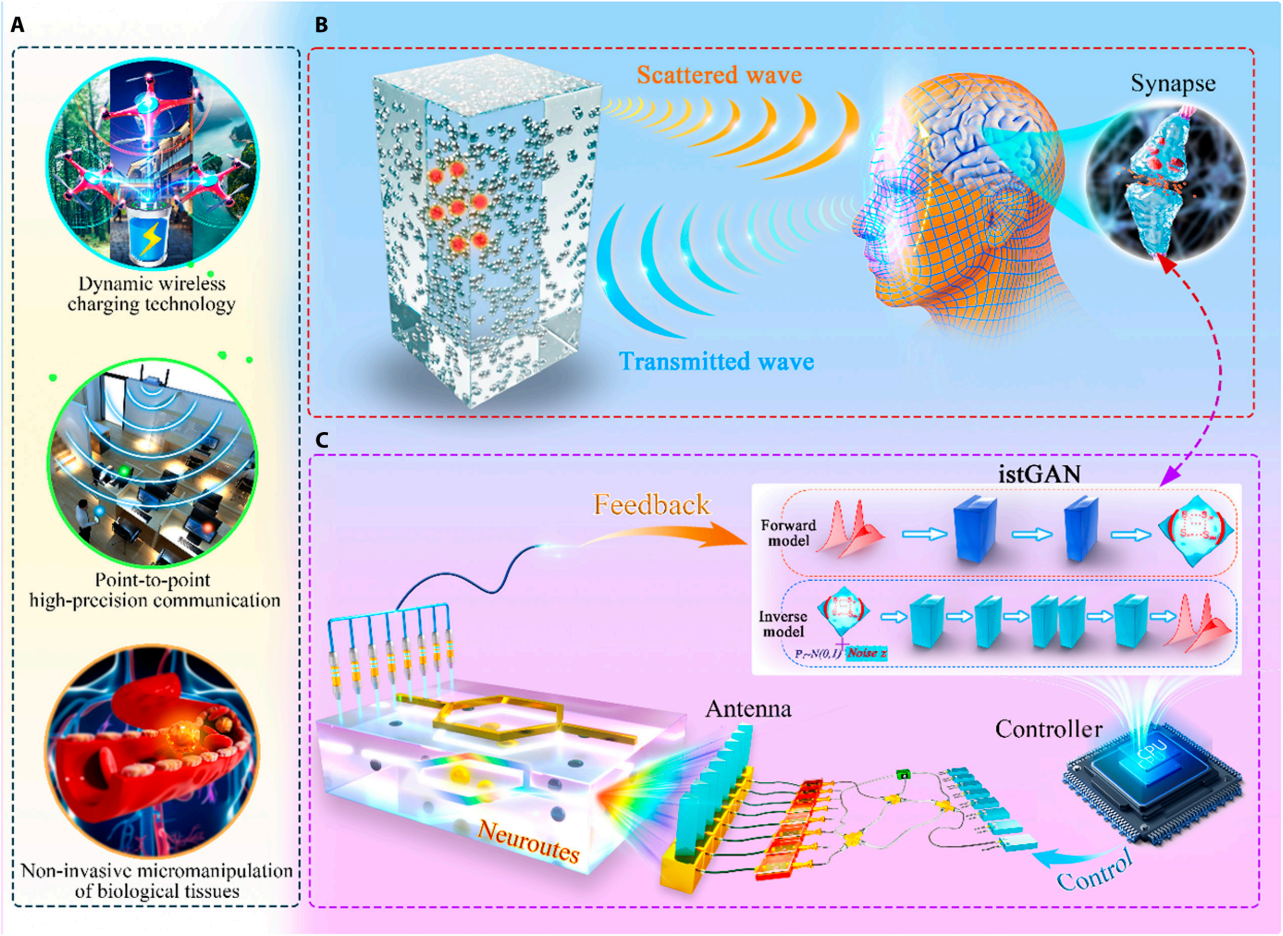
Illustration of the Neuroute concept inside dynamic scattering spaces. (A) Various applications, including dynamic wireless charging technology, point-to-point high-precision communication, and non-invasive micromanipulation of biological tissues, pose stringent demands for efficient information states’ acquisition inside dynamic scattering spaces. (B) The retina of human eyes transmits the perceptual information to neurons, and the nervous system controls the ciliary muscles for self-adaptive adjustment. Reversibly, when self-adaptive information states’ acquisition is carried out to multi-targets (shown by the red dot) in scattering spaces, the scattered wave can be analyzed by artificial synapses, and the transmitted wave can be controlled by the tunable perspective module. (C) Analogously, a controller embedded with an artificial neural network can be utilized to shape the wave field propagation routes by synthesizing scattering spaces, thus creating a neural route capable of transmitting information states on demand in a non-invasive and self-adaptive manner. This innovative approach is referred to as the Neuroute.

The physical essence of information states involves parameters’ precise estimation or measurement, serving as the foundation for all coherent measurement scenarios. As depicted in Fig. 1A, some quintessential dynamic scattering spaces are revealed, including indoor point-to-point communication, wireless charging of unmanned aircraft systems, and the micromanipulation of biological tissues. A common feature of these scenarios is that the wave field routes are interfered by dynamic scattering spaces, rendering the non-invasive information states’ acquisition seemingly unattainable.

In addressing this challenge, the Neuroute leverages the principles of human information acquisition. We delineate the inverse process of human information acquisition, as depicted in Fig. 1B: Humans gather the wave field information reflected from the scattering space for analysis and assessment, enabling the dynamic adjustment of transmitted waves to acquire information states for multiple targets inside scattering spaces. This process bears resemblance to the functionality of perspective eyes [46].

As illustrated in Fig. 1C, to intelligently assign the desired information states to multi-targets inside dynamic scattering spaces, the controller incorporates a neural network that emulates human brain neurons. The physical input to the neural network comprises solely external scattering matrix without any local information, facilitating rapid excitation of the Neuroute generator. Subsequently, the Neuroute demonstrates the capability for self-adaptive information states’ acquisition for multi-targets following a single non-invasive measurement, whether in the actual scattering space in Fig. 1A or the equivalent black box in Fig. 1B.

### Experimental verification of the Neuroute

**Fig. 2.**
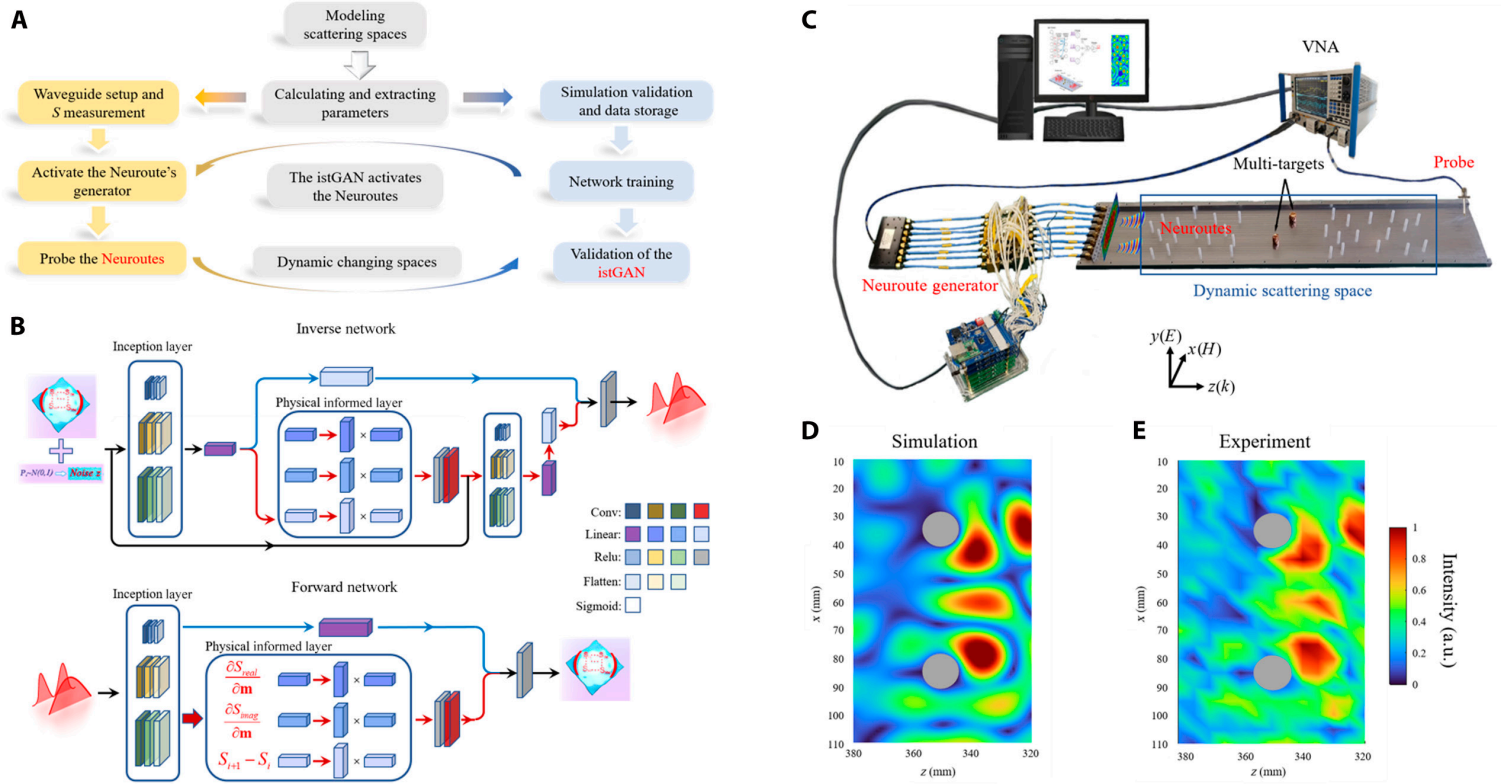
Experimental verification of the Neuroute embedded with the istGAN. (A) A simplified flowchart for generating the Neuroute comprises 2 primary components: the construction of the istGAN and experimental validation. (B) The istGAN architecture schematic. Considering the one-to-many mapping between the scattering matrix and the on-demand wavefront, we divide istGAN into forward and inverse models, and the convergence condition depends on cyclic iterations of the 2 networks. The istGAN is specially designed for non-invasive self-adaptive information states’ acquisition inside scattering spaces, drawing on the principle of the cGAN and the tandem neural network. (C) Experimental setup. (D) The single-target Neuroute for information states’ acquisition is simulated by utilizing a sole measurement of the scattering matrix. See Movie S1 for detailed information about processing. (E) Corresponding experimental results using equipment in (C).

To evaluate the effectiveness of the Neuroute, we employ a framework that integrates physical experiments with intelligent algorithms, as depicted in Fig. 2A. At the core of this framework lies the construction of istGAN, based on the assumption of modeling the scattering space. We equate this space to an environment comprising random scatterers (refer to Fig. S1) and extract essential parameters using numerical simulation methods.

In our numerical simulation, while the Neuroute can be seamlessly integrated with nearly any matrix or operator, for convenience, we have modified the generalized Wigner–Smith (GWS) operator [40] for multi-targets to construct a mapping dataset between the Neuroute morphology and the scattering matrix of dynamic scattering spaces. However, the presence of random scattering poses significant challenges to full-wave simulation, hindering the extraction of the scattering matrix **S** and the use of the GWS operator **Q***_α_* ∝  − *i***S**^−1^Δ**S**. To address this, we propose the use of group *T*-matrix to perform quasi-static discretization and wave field construction inside dynamic scattering spaces (see Materials and Methods and Note S2 for more information). Additionally, the mutual coupling characterization between the excitation elements will greatly increase the response time of the Neuroute and make it unable to adapt to the dynamic scattering spaces. To circumvent this, we have adopted an efficient method based on characteristic mode theory to conceptually extend the front excitation vector (see Materials and Methods and Note S3).

The aforementioned efficient numerical simulations serve as a foundation for the istGAN shown in Fig. 2B, where a pre-trained forward surrogate network replaces the discriminator in traditional generative adversarial networks, for evaluating the data quality from the generator shown as the inverse network. To emulate the characteristics of the convolutional kernel in the convolutional neural network and effectively extract features from the input data, the sizes of the 3 filters (represented as cuboids in Fig. 2B) within the inception layer of istGAN are different, *k* = 3, *k* = 5, and *k* = 7, respectively, where *k* denotes the filter size. To preserve the spatial dimensions of the output, zero-padding is essential for the input matrix (also see Materials and Methods and Note S4 for more information). Thus, our istGAN mainly includes 2 networks. One network is the forward surrogate network, which models the mapping from information states on demand **ψ**_demand_ to the **S** matrix, incorporating the inception layer to fully extract input data features and the physical informed layer to analyze the unitary components of the external scattering matrix. The other network is the inverse generation network, which fits the **ψ**_demand_ statistically through the Gaussian noise and **S** matrix, inspired by the conditional Generative Adversarial Network (cGAN). Furthermore, within the istGAN, the **S** matrix can be regarded as an 8 × 8 2-dimensional matrix, and the introduced Gaussian noise also exists as a 2-dimensional matrix of identical dimensions. To effectively capture the gradient information of the **S** matrix, a physical-informed layer is incorporated into the inverse generation network. This layer encompasses the **S** matrix gradient information of real and imaginary parts ($\frac{\partial S_{\text{real}}}{\partial m}$ and $\frac{\partial S_{\text{imag}}}{\partial m}$, respectively, where **m** denotes the input plane) as well as the difference of the **S** matrix *S*_*i*+1_ − *S_i_* in the time sequence domain. This integration empowers the istGAN to non-invasively assimilate local spatial gradient information during the dynamic evolution of the **S** matrix. For further details, please refer to Note S7. The output result error of the inverse generation network is assessed by the forward surrogate network.

As shown in Fig. 2C, we perform the proof-of-concept verification in the microwave frequency of 10 GHz. Firstly, as described in Fig. S4, a rectangular waveguide is used as the external environment supporting *N* = 10 transverse modes, while the scattering space is equivalented by randomly placing 30 Teflon scatters (optionally, mixed with 4 copper scatterers) with a radius of 1.5 mm. Additionally, 2 metal scatters with a radius of 5.5 mm are employed to represent multi-targets awaiting information states’ acquisition. Moreover, the istGAN embedded in an upper computer that acquires signals sampled by the electric array probes and read by the vector network analyzer (VNA) excites the Neuroute through the Neuroute generator (mainly composed of a multi-layer microprogrammed control unit, a power divider, 8 phase shifters, and 8 attenuators). Furthermore, a buffer of 100 mm is created on both sides to mitigate other components caused by the antenna’s near field (see Materials and Methods and Note S5 for more information).

Following the integrating of istGAN, the Neuroute for multi-target information states’ acquisition no longer necessitates local information Δ**S**, omitting intrusive adjustments and operational iterations. In essence, only a single measurement of the external scattering matrix is required, rendering the process non-invasive and self-adaptive. To ascertain whether the Neuroute can achieve the same effect as the traditional wave field routes more efficiently, we compared the process of generating wave field routes with the classical GWS operator and the process of generating the Neuroute (see Movie S1), validating the efficiency and consistency. Additionally, taking a given scattering space as an example, the Neuroute morphology corresponding to the istGAN output excitation is calculated, as shown in Fig. 2D, which aligns well with the Neuroute morphology (Fig. 2E) measured using equipment in Fig. 2C.

### Neuroute inside dynamic scattering spaces

The functionality of Neuroute as described in the provided text involves 2 key points: convergence in a non-invasive environment and self-adaptability inside dynamic scattering spaces.

**Fig. 3.**
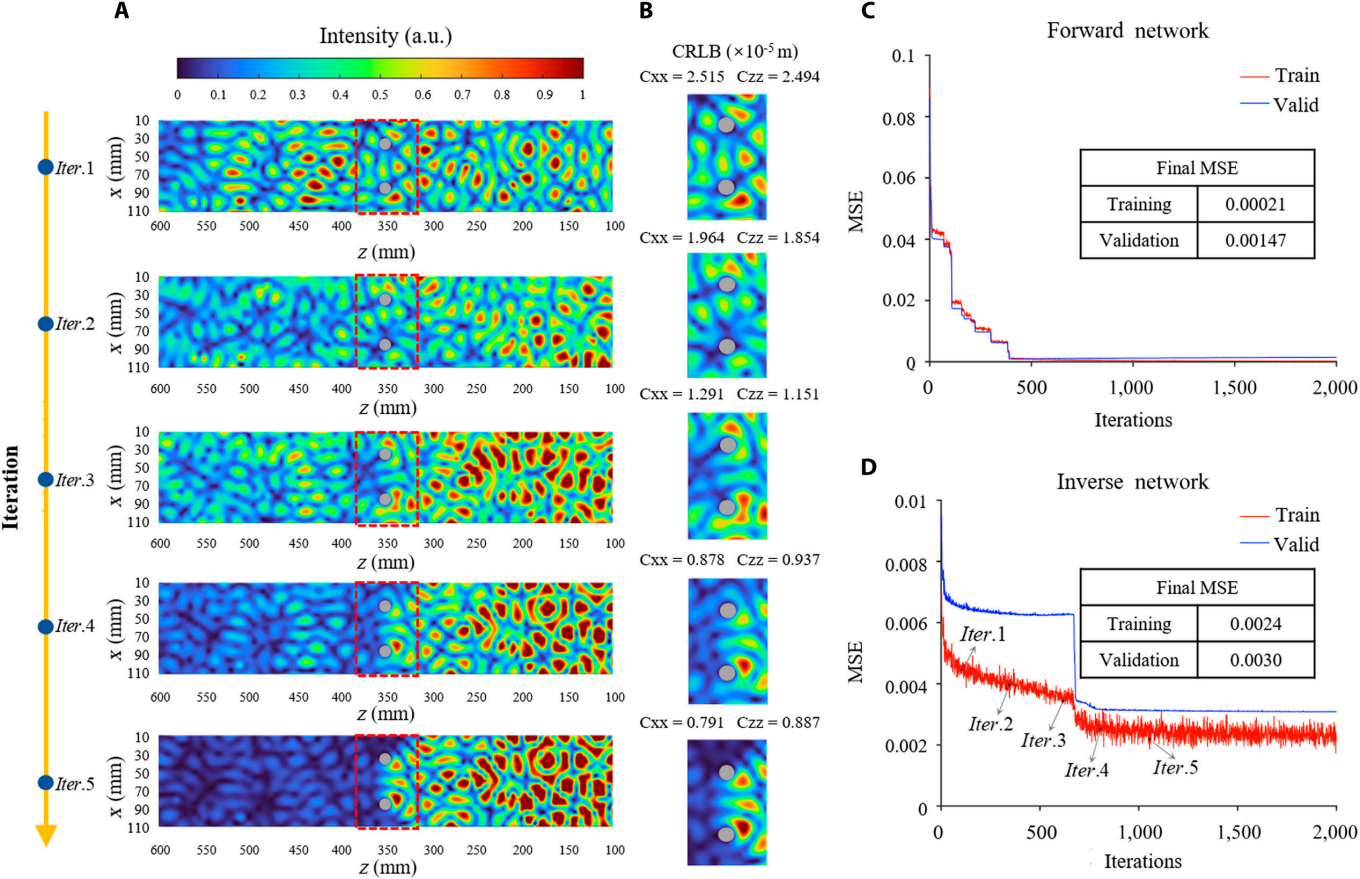
Experimental proof of convergence of the Neuroute embedded with the istGAN. (A) The morphological evolution of the Neuroute at different stages prior to convergence is depicted. Operationally, the istGAN is embedded in the experimental device at different training stages, and the Neuroute generator is regulated according to the given phase output, aligning with the experimental outcomes of the multi-target area as illustrated in (B). The normalized electric field distribution surrounding the local area of multi-targets with the CRLB parameters are employed to visually observe the convergence property of the istGAN. (C) Loss function of the forward model of the istGAN; the average training mean square error (MSE) converged to less than 0.00021 after 2,000 steps. (D) Loss function of the inverse model of the istGAN based on the pre-trained forward model; the average training loss converges to less than 0.0024 after 2,000 steps.

The convergence of Neuroute is demonstrated through the convergence of information states near multi-targets with a single non-invasive measurement. This is achieved by intercepting 2*λ* wave field routes along the *z* direction. The convergence is visually shown in Fig. 3A, while the corresponding information states near the multi-targets are depicted by the local electric field pattern shown in Fig. 3B. Evidently, the information states near the multi-targets exhibit strong convergence with a single non-invasive measurement. Considering that the Neuroute essentially originates from the local momentum shift Δ**k***_i_* caused by the interaction with *i*th target [23], we conduct vector superposition of different ∑Δ**k***_i_* in our experiment to obtain the on-demand information states of multi-targets (see Note S6 for more information). This might appear counterintuitive in cases of measurements not involving information inside the scattering space, but in fact the local information inside the scattering space is contained in the pre-trained network (see Note S7 for more information). As depicted in Fig. 3C and D, we present the mean square error (MSE) alongside the number of iterations of the forward network and inverse network in the istGAN, respectively, demonstrating the reliability and convergence of our pre-trained network in revealing features inside scattering spaces. Moreover, the inclusion of multi-frequency information will enrich the available **S** data, thereby aiding the istGAN in more effectively acquiring the frequency-independent mapping between **S** and the operator **Q***_α_* ∝  − *i***S**^−1^Δ**S** when the existing **S** data are inadequate. However, in the generation of our dataset, we ensure the generation of ample **S** data by manipulating 15,000 scatterer configurations; hence, only single frequency input is required.

**Fig. 4.**
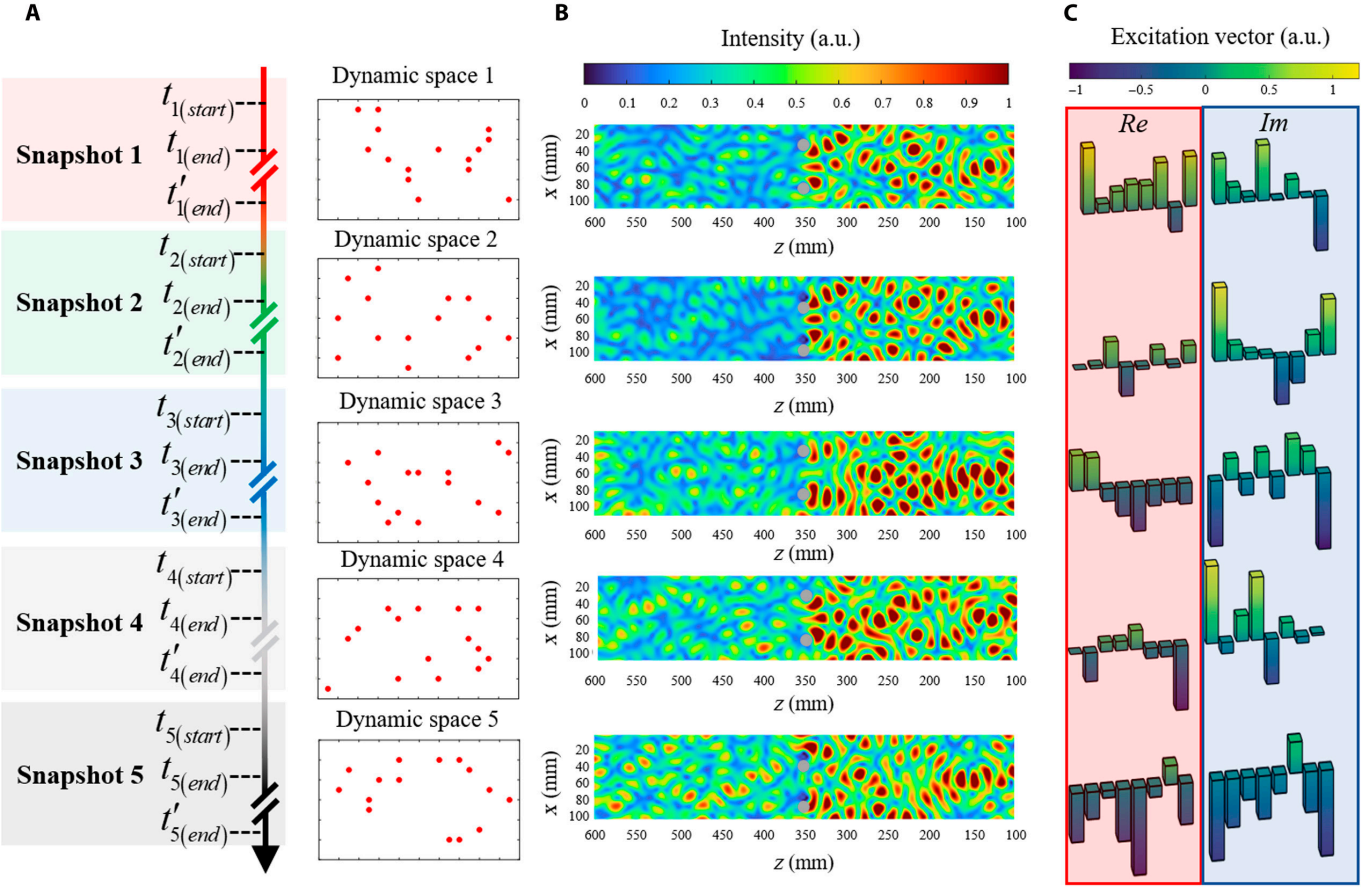
Experimental proof of universality of the Neuroute inside dynamic scattering spaces. (A) Schematic diagram of the scatterer distribution inside each scattering space, with each red dot representing a scatterer. (B) The Neuroute morphology corresponding to the 5 scattering spaces is delineated by the detected electric field intensity distributions. The information states’ acquisition near multi-targets (depicted by the gray circle in the figure) demonstrates exceptional performance. (C) Excitation distributions of 5 scattering spaces recorded by the Neuroute generator. The red box is the excitation amplitude of the 8 ports, whereas the blue box represents the excitation phase.

To verify the self-adaptability of the Neuroute, we select 5 snapshots of scatterer distribution along the time axis to quasi-statically present the dynamic scattering environment, as illustrated in Fig. 4A. On this axis, *t*_*i*(*start*)_(*i* = 1, 2, …, 5) denotes the moment when the scattering matrix of the *i*th scattering space is measured, *t*_*i*(*end*)_ denotes the moment when the *i*th control of the Neuroute generator is completed, and $t_{i\left(\mathrm{end}\right)}^{\prime }$ denotes the moment when the *i*th scattering space’s electric field measurement is completed and the next scattering space configuration is formed. Due to the exceptional efficiency of the Neuroute system, the time between *t*_*i*(*start*)_ and *t*_*i*(*end*)_ is measured in milliseconds. In Fig. 4C, the excitation results of generating the corresponding Neuroute given by the istGAN are depicted. As our experiments are stimulated by 8 excitation ports, each frame comprises 8 complex points constituting the excitation vectors. In proportion to the excitation vectors of the corresponding Neuroute morphology given by the istGAN, the Neuroute generator excites the information states on demand at this moment (also anti-transmission, see Note S8 for more information). The Neuroute morphology of the 5 selected snapshots is shown by the normalized electric field patterns in Fig. 4B. The results underscore the Neuroute’s capacity to precisely acquire information states inside dynamic scattering environments. Further cases showcase the applicability of the istGAN across various scenarios (refer to Note S8), encompassing frequency shifts, alterations in scatterer shape and quantity, and the emergence of clustered scatterers.

### Toward the Fisher information limit using the Neuroute

The above sections have elucidated the construction of the Neuroute embedded with the GWS operator using our proposed methodology for fast, adaptive, and non-invasive acquisition of information states inside dynamic scattering spaces. Subsequently, our focus shifted to quantifying the parameter estimation precision limit using the Neuroute.

Different scattering configurations have varying Neuroute morphologies. As we dynamically adjusted scattering spaces, we observed that the information states were significantly influenced by the degree of scattering. This is primarily due to the impact of the mean free path of the scattering space on wave field transmission. Enhancing the degree of scattering, or even inducing Anderson localization, substantially constrains the effectiveness of information states’ acquisition by the Neuroute. The Fisher information matrix furnishes an objective function for quantifying information states. However, owing to its reliance on field distribution, it necessitates numerous forward calculations and iterations for limit determination, rendering it extremely time-consuming. The Neuroute introduces a novel methodology for efficiently characterizing the parameter estimation precision limit in a statistical sense.

**Fig. 5.**
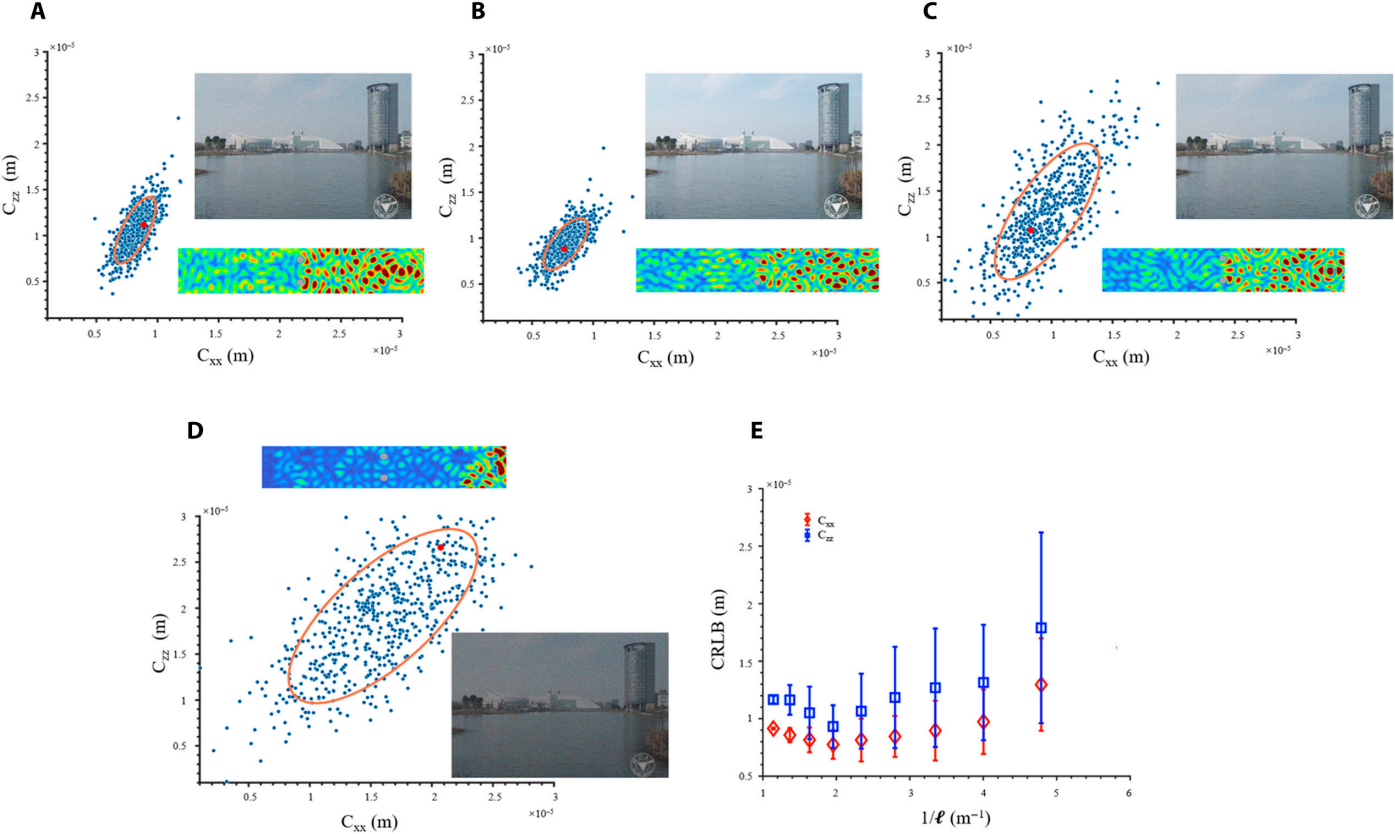
Toward the Fisher information limit using the Neuroute. The Cramér–Rao lower bound (CRLB) pertaining to the Neuroute information states is presented. Additionally, the Neuroute morphologic patterns associated with the red dot and the images’ multiplexing transmission effects corresponding to the ellipses are depicted. (A) The CRLB distribution in the case of 1/*l* = 1.4 m^−1^, corresponding to the scenario with extremely small scattering, where *l* represents the mean free path of the wave field. (B) The CRLB distribution in the case of 1/*l* = 2 m^−1^, corresponding to the scenario with optimal scattering. (C) The CRLB distribution in the case of 1/*l* = 4 m^−1^, corresponding to the scenario with large scattering. (D) The CRLB distribution in the case of 1/*l* = 5.8 m^−1^, corresponding to the scenario with extremely large scattering tending to the Anderson localization. (E) The relationship between CRLB of 2 directions and the degree of scattering 1/*l*. Each point denotes the geometric mean of 700 corresponding Neuroute tested for each scattering degree, and error bars represent 1-sigma intervals.

To expound on the method for quantifying the parameter estimation precision limit using the Neuroute, we still employ the GWS operator for data construction. Figure 5A to D depict 4 Fisher information features with the Cramér-Rao lower bound (CRLB) scheme (also see Materials and Methods and Note S9), corresponding to scattering spaces with different degrees of mean free path. The comprehensive quantization effect of information states can be observed through the dense scatter plot distribution, where different points represent distinct distributions of scattering configurations under the same mean free path degree (see Materials and Methods). The Neuroute corresponding to the 4 red dots is illustrated by the small figures within Fig. 5A to D, representing weak scattering (Fig. 5A), medium scattering (Fig. 5B), strong scattering (Fig. 5C), and a scattering space tending toward Anderson localization (Fig. 5D), respectively. The CRLB value of information states is a crucial parameter reflecting the quality of parameter estimation. Wireless image transmission and video transmission conducted based on corresponding *C*xx and *C*zz can effectively demonstrate the statistical properties of information states in dynamic scattering environments, as depicted in Fig. 5A to D and Movie S2. For detailed methods of generating images and videos, please refer to Materials and Methods. In Fig. 5E, 10 scenarios are expanded, and their Fisher information characteristics are fitted. The increase in the scattering degree inside the scattering space demonstrates a trend of first increasing and then decreasing the Fisher information limit of this scene. The average free path corresponding to the Fisher information peak point is approximately equal to the longitudinal propagation distance of the scattering space. Using the Fisher information matrix as the objective function, we conduct optimization with a heuristic algorithm, and obtained the maximum information states quantity for 10 scenarios, as depicted in the blue curve in Fig. S8, which is in good agreement with Fisher information curve obtained by the Neuroute embedded with the GWS operator. In conclusion, our work represents the first successful achievement of efficiently reaching the Fisher information limit using Neuroutes, which paves the way for constructing neural statistic dynamics, including generation, classification, and deriving the limit efficiently.

## Discussion

To summarize, the Neuroute significantly enhances the information states’ acquisition efficiency with the istGAN, effectively addressing non-unique mapping problems inside dynamic scattering spaces and uncovering more novel physical phenomena. This innovation presents a radically new information states’ acquisition mechanism within our proof-of-concept experimental system, holding promise for a wide range of future applications involving high-density targets. While recent works have realized basic coherent wave effects [19,30,47,48], they have lacked non-invasive intelligent solutions, limiting their practical use. It is astounding to see that the Fisher information limit can be reached even more efficiently without any partial incursion. By integrating the Neuroute methodology and classical micro–nano manipulation techniques such as the millimeter-wave photonic limiter [49] and phase change materials [50], our concept can be extended to millimeter-wave, terahertz, and optical bands, thereby pushing the boundaries of information states’ acquisition to new horizons and demonstrating a radically new information states’ acquisition mechanism with a myriad of future applications.

Looking to the future, it is intriguing to contemplate the potential for advanced information acquisition systems to function inside increasingly intricate scattering spaces, facilitated by Neuroute’s capacity to communicate physical information through scientific neural networks [51,52]. An additional valuable enhancement involves the intelligent Neuroute generation method, employing on-site learning to adapt to a more conventional open-loop operating system [53], thereby enhancing its resilience to unforeseen stimuli. In terms of hardware configurations, combined with high-speed control advantages of parallel computing [54] and optical control, the Neuroute will have a lower response time and stronger processing power. Furthermore, the inverse engineering process involved in simulating the physical mechanisms of human eyes revitalizes the design of bionic systems. If additional designs are developed in tandem with physical symmetry, the synergies between human and natural intelligence hold exciting potential.

## Materials and Methods

### Numerical modeling and data collection

This section furnishes specific numerical dataset details pertinent to the Neuroute, forming the foundation for its efficient adaptation to dynamic scattering spaces. The scattering space is modeled as a 2-dimensional form of the Helmholtz equation $\begin{array}{c} \nabla \times \nabla \times \psi \left(r\right)-k_{0}^{2}\varepsilon \left(r\right)\psi \left(r\right)=\mathrm{i\omega}\mu_{0}J_{\mathrm{inc}} \end{array}$, where ***J****_inc_* denotes the current distribution, *ψ*(**r**) signifies the electric field defined inside scattering spaces, *k*_0_ represents the wave number, and *ε*(**r**) encapsulates the spatially varying dielectric constant.

Given the substantial presence of numerous scatterers in our model, conducting numerical simulations using conventional full-wave numerical analysis software poses significant challenges. Consequently, we employ the group-*T*-matrix method to effectively formulate the electric field distribution inside the waveguide, based on the following equation:

$$T_{m}^{\left(p\right)}=\frac{k_{p}J_{m^{\prime }}\left(k_{p}a_{p}\right)-J_{m}\left(k_{p}a_{p}\right){\mathrm{kJ}}_{m^{\prime }}\left({\mathrm{ka}}_{p}\right)}{{\mathrm{kH}}_{m}^{\left(1\right)}\left({\mathrm{ka}}_{p}\right)J_{m}\left(k_{p}a_{p}\right)-H_{m}^{\left(1\right)}\left({\mathrm{ka}}_{p}\right)k_{p}J_{m^{\prime }}\left(k_{p}a_{p}\right)},$$ (1)

where *T*^(*p*)^ is the group-*T*-matrix representing the conversion effect of the *p*th clustered scatterer, giving the scattering matrix **S** numerical solutions, *k* is the wave number and *k_p_* is the inner wave number of the *p*th scatter, *J*_n_ is the Bessel function of order *n*, and $H_{n}^{\left(1\right)}$ is the Hankel function of order *n*. This approach enables the efficient characterization of the wave field and information states inside diverse scattering spaces, facilitating data collection (see Note S2 for details).

Furthermore, to mitigate potential distortion of the Neuroute induced by mutual coupling, we employ a coupling compensation method to efficiently characterize the wave port, as given by:

$${\left[W_{c}\right]}_{i}=\sum_{i=1}^{\mathrm{MN}}\sum_{m=1}^{\mathrm{MN}}{\left[I_{\lambda }\right]}_{i}Q\left(i,m\right){\left[W_{0}\right]}_{m},$$ (2)

where **W***_c_* denotes the excitation vector considering mutual coupling, generating an electric field $E=\sum_{i=1}^{\mathrm{MN}}{\left[\mathrm{AF}\right]}_{i}{\left[W_{c}\right]}_{i}$ applied to shape the Neuroute with array factor without coupling **AF**. *M* is the mode number supported by an element while N is the element number. Moreover, **Q** represents the compensation matrix detailed in Note S3.

Consequently, the training data are obtained through our efficient algorithm. For the scattering matrix **S**, 15,000 configurations of scattering spaces have been computed to simulate various real-life environments. Subsequently, each **S** obtains the corresponding excitation vector ***J****_inc_* according to the GWS operator, serving as the label for information states’ acquisition. All data collection processes are implemented in MATLAB, and the accuracy of the numerical computation results is validated by Lumerical FDTD and Altair FEKO.

### Training of the Neuroute

The Neuroute is trained using Python version 3.9.12 and torch framework version 1.12.1 on a server (NVIDIA TITAN Xp GPU and AMD Ryzen Threadripper 1950X 16-Core Processor with 128 GB RAM, running on a Linux operating system). It takes a few minutes for the Neuroute to converge, but one can get a millisecond response when using it offline.

### Experimental setup for the Neuroute validation

An aluminum alloy waveguide, with dimensions of 100 mm inner width along the *y* direction, 110 mm inner height along the *x* direction, and 700 mm length along the *z* direction, is fabricated to support 10 transverse electric field modes. This waveguide is augmented with 2 distinct types of scatterers, namely, 2.5-mm-radius Teflon cylinders and 5.5-mm-radius copper cylinders, to emulate the scattering phenomena. Multi-targets are positioned inside the central region of the waveguide, awaiting the Neuroute generation and information states’ acquisition. Custom-designed excitation array antennas and electric field probes are positioned at the front end and the back end of the waveguide, respectively. The excitation antenna is connected to the Neuroute generator, while the electric field probe is connected to the VNA to deliver data to the upper computer. The Neuroute generator comprises a multi-layer microprocessor integrated with the istGAN, phase shifter, and attenuator to make intelligent decisions. It is important to note that these devices are utilized solely to demonstrate the information states’ acquisition in the microwave band and do not influence core components of the Neuroute.

As the Neuroute generation depends on the real-time scattering matrix **S**, 8 ports on each side of the waveguide are scanned by VNA with a switch and measured according to *S*[*m*, *n*] = *S_mn_* = *S_nm_* (*m* is the input port and *n* is the output port). Additionally, to account for the diffusive regime of our model, the mean free path *l* of the current scattering space can be measured according to ⟨*T*⟩ = *l*/*L* to characterize the scattering degree, where *L* denotes the transmission length, and ⟨*T*⟩ is the average transmission of the wavefront contributed by $T=\sum_{m,n=1}^{N}{\left|S_{\mathrm{mn}}\right|}^{2}/N$; *N* = 8 represents the number of controllable modes. It is noteworthy that the selection of *N* = 8 is deliberate, as the number of controllable modes is intentionally set to be less than the number of waveguide modes due to the substantial quantity of modes within the actual scattering space.

Upon completion of the Neuroute morphology calculation, our system will sensibly provide the excitation vector. Experimentally, it is necessary to adjust the numerical settings of the electronic control phase shifter and electronic control attenuator in accordance with the excitation vector, a process also managed by the multi-layer microprocessor. To validate the successful operation of the Neuroute, we have introduced 5 mm × 5 mm uniform holes in the upper metal plate of the waveguide. These holes assist in the reconstruction of the internal electric field with the aid of a probe during the verification process. To mitigate the electromagnetic radiation stemming from the perforations, non-test holes will be shielded using tin foil throughout our testing procedures.

### Toward Fisher information limit and emulation of images and video transmission with the Neuroute experimentally

Once the desired excitation vector is determined, the Neuroute can be efficiently generated experimentally, and the corresponding electric field distribution in scattering spaces can be measured. Here, we give a method to characterize the parameter estimation precision limit on this basis.

The Neuroute is adept at non-invasively acquiring information states by focusing on the local features of multi-targets rather than the overall features of scattered spaces. Considering the electric field distribution *E_θ_* associated with the unknown parameter *θ* and sample points *N*_m_ around the *m*th target, with a total of *N* multi-targets, we can obtain the Fisher information matrix *I*, given by:

$$I_{\mathrm{ij}}\left(\theta \right)=\sum_{m=1}^{N}\sum_{k=1}^{N_{m}}\frac{1}{E_{\theta }\left(k\right)+\beta }\left(\frac{\partial E_{\theta }\left(k\right)}{\partial \theta_{i}}\right)\left(\frac{\partial E_{\theta }\left(k\right)}{\partial \theta_{j}}\right)$$ (3)

where *β* denotes the additive Poisson noise caused by our measurements. Furthermore, the CRLB $C_{i}=\sqrt{{\left[I^{-1}\left(\theta \right)\right]}_{\mathrm{ii}}}$ is employed to characterize a given Neuroute.

The parameter estimation precision limit is depicted through scatter plots comprising 700 configurations for each scattering degree. For the same degree of mean free path *l*, the scattered points will form an ellipse with a given standard error. The center and axis length of the ellipse represent the parameter estimation precision limit expectation and variance, respectively. The comparison results in Fig. 5E are realized by using a genetic algorithm in the MATLAB toolbox to verify the speed and accuracy of the Neuroute.

For Fig. 5A to D, images are discretized into a sequence of binary pixels using the binary phase shift keying scheme. Each pixel is represented by 3 integers ranging from 0 to 255, corresponding to 8 bits, in accordance with the RGB color model. The mean and variance of 700 Neuroute configurations, as determined experimentally, correspond to the signal transmission intensity and variance of external Gaussian white noise, respectively. This configuration establishes a classic case of signal multiplexing transmission. For Movie S2, each frame of the video corresponds to a specific point in Fig. 5A to D, and the remaining data undergo processing analogous to that of image transmission. In contrast to the examination of Neuroute’s statistical properties in image transmission, the emphasis here lies in scrutinizing Neuroute’s evolution inside a dynamic environment.

## Data Availability

All data needed to evaluate the conclusions in the paper are present in the paper and/or the Supplementary Materials.
