# Supplementary material for: Non-Invasive Self-Adaptive Information States’ Acquisition inside Dynamic Scattering Spaces
Source: Research (Wash D C). 2024 May 31;7:0375. doi: 10.34133/research.0375 (PMC11140760; doi:10.34133/research.0375)
Supplement: Supplementary 1 — Figs. S1 to S8 Table S1 Movies S1 and S2 [file research.0375.f1.zip › 0. RESEARCH_SM.f2.docx]

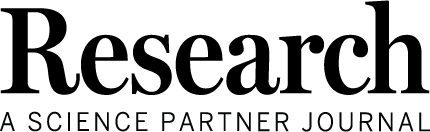


Supplementary Materials for

**Non-Invasive Self-Adaptive Information States Acquisition inside Dynamic Scattering Spaces**

Ruifeng Li *et al.*

*Corresponding author. Email: [liep@zju.edu.cn](mailto:liep@zju.edu.cn)

**This PDF file includes:**

Supplementary Text

Figs. S1 to S8

Tables S1

Movies S1 to S2

References (1 to 9)

Supplementary Text

***1. Physical model and mechanism of scattering spaces***

The scattering space can be modeled in a variety of convenient ways. In this section, we focus on a physical model that simulates the scattering space by means of scatterers placed in waveguides. In principle, the scattering degree is determined by the density of scatterers and the scattering cross section 1:

, (S1)

where **S**inc denotes the incident energy flux, **S** represents an arbitrary surface enclosing scatters, **E**s the scattered electric field, and **H***s the complex conjugation of the magnetic field. Essentially, for our model, taking a single scatterer at a given incident wavelength , depends only on the scatterer radius *r* and dielectric constant , shown in Fig. S1. Hence, the variety of the scattering degree is simulated in our experiment by different and , and quantized by the mean free path obtained according to the Helmholtz equation.

For quantitative characterization, we use the scattering mean free path *l* to reflect the scattering degree2:

. (S2)

Considering that the scatterers used to describe the multi-targets will also be coupled to the surrounding environment, we use the modified electrical polarization to describe the feature of multi-targets3:

, (S3)

where is the polarizability of *i*th targets, *k* is the wave number, and represents the Green function difference with and without the scattering space. Apparently, for weakly coupling cases.

For experimental validation, the piecewise test of transmission rate is an effective method to verify the constructed scattering space4:

, (S4)

where *L* denotes the transmission length, the test process of total *T* can be seen in Methods.

***2. Electromagnetic simulations of scattering spaces***

When employing commercial full-wave software for the numerical resolution, the computational simulation becomes exceedingly time-consuming, primarily owing to the intricate multi-scale phenomena and the presence of multiple scattering issues. This limitation significantly impedes the expeditious acquisition of data. In this section, we employ a characterization method based on group-*T*-matrix to facilitate the extraction of scattering matrix *S* correlation terms in the GWS operator.

Considering the coupling between scatterers, we decompose the waves on the *q*th scatterer into the superposition of incident waves and coupled waves 5:

. (S5)

The current on each port of the Neuroute generator can be expanded as superposition of dipole current, and can be expanded as:

, (S6)

where is the Hankel function of order *n*=0. Following the same form, can be expanded into:

, (S7)

where is the Bessel function of order *n*=0. Then the relationship between the incident wave and the scattered wave is established by the group-*T*-matrix method:

, (S8)

it can be seen in Methods for more information about the group-*T*-matrix . Comparing coefficients of the above equations can efficiently solve the wave field distribution in scattering spaces.

When it comes to the GWS operator , the global feature **S** and local feature can be easily obtained from :

. (S9)

Our previous work has shown the high efficiency of the method for electromagnetic simulations of scattering spaces.

***3. Efficient characterization of the Neuroute generator***

The ideal independent ports are difficult to achieve in practice. To generate the pure Neuroute, we need to fully characterize the coupling between the ports of the Neuroute generator based on the element theory of characteristic mode (ETCM).

Considering two ports named ‘A’ and ‘B’, the mode excitation coefficient of the port ‘A’ is constructed by the origin electric field and the coupled electric field :

, (S10)

where denotes the *n*th mode current on ‘A’, and T is the transpose operation. Considering mode decomposition, electric field components can be expressed as with the total number of modes *M*, where with the eigenvalue of the *n*th modes. Therefore, we can extend the electrical properties of multiports considering coupling to the matrix form6, 7:

, (S11)

where **W** is an unknown matrix representing the excitation coefficient of each mode current at each port, and the remaining matrix is represented by mode current and mode electric field as follows:

. (S12)

For solving , a modified matrix considering the coupling can be added into a traditional array factor, shown in Methods. Fig. S2 and Table. S1 show the accuracy and efficiency of our method respectively.

***4. Architecture and training process of the istGAN of the Neuroute***

The istGAN is proposed to achieve information states acquisition on demand (also known as generating the Neuroute) based on the CycleGAN framework. The detailed network architecture of the istGAN is shown in Fig. S3.

For the training process, 15000 samples are collected and divided into a training set (13500 samples) and a validation set (1500 samples). Among them, the gradient evolution and weight optimization of the istGAN are mainly realized with the help of training sets, and the istGAN can be used to generate the Neuroute after being calibrated by the verification set. The input information of the istGAN mainly consists of the on-demand wave field state demand and the scattering matrix **S**, which are respectively fed into two parts of the istGAN. To facilitate convergence, the data are normalized.

For the physical informed layer shown in Fig. 2B, it adopts the fully connected structure that takes the reshaped scattering matrix **S** formed *N***N*0*2 as input, where *N* denotes the number of ports and *N*0 denotes the batch size, comprising the mean free path 1/*l*. When it comes to the inverse network, we take the direction vector as input to obtain the local matrix composed of as we notice that spatial gradient information has been lost in our Neuroute framework. After several iterations, the istGAN can learn the spatial gradient information of the scattering matrix, so the steep drop property in the inverse network appears in Fig. 3D. More physical explanations can be seen in Supplementary note S7.

Specifically, the input of the forward surrogate network is demand, which contains amplitude and phase information, and the output is the scattering matrix **S**. The objective function of the istGAN draws on the conditional generation adversarial network (cGAN):

(S13)

where *V* is cross entropy, *G* generator, and *D* discriminator. represents the fake data generated in the generator based on *z* according to condition *c*, while represents the result judged by the discriminator based on *z* according to condition *c*. It is worth noting that the istGAN is naturally suitable for solving the non-unique mapping problem discussed in this paper, whether it is fixed one component to train another or simultaneously.

More importantly, in the inverse generation network, the noise with Gaussian distribution and the scattering matrix **S** as constraints are input into the network, and the generated by the istGAN contains amplitude and phase information. Then, the generated are sent into the pre-trained forward surrogate model to obtain the scattering matrix **S** corresponding to . The loss of network is evaluated by the mean square error function to adjust network parameters:

(S14)

where *N* is the sample number of each batch, denotes the output value of the forward surrogate model, and represents the value of the scattering matrix **S** in our data sets.

***5. Experimental setup and measurement***

To reduce the quantization difficulty of the Neuroute, our experiments are carried out in a rectangular waveguide supporting only transverse electrical modes, as shown in Fig. S4a. The probe shown in Fig. S4b is attached to a VNA, embedded with ports scanning and data analysis program that provides the scattering matrix information to the Neuroute generator controller shown in Fig. S4c. In addition, in the stage of data set construction, we also need to perturb the position of the multi-targets to test the change of the scattering matrix . Besides, to avoid the near-field components of the electric field generated by the eight ports, the internal scatterer region is kept at least 100mm apart from the port. The near-field attenuation rule is shown in Fig. S5, which is the verification of the rationality of the experimental setting.

***6. Physical mechanism of the multi-target information states acquisition***

Considering that we demonstrate the Neuroute’ performance with the help of the GWS operator, in this section we continue to take the GWS operator as an example to illustrate the physical mechanism of multi-target information states acquisition.

For multi-target information states acquisition, the core is capturing the physical parameters that determine the distribution of information states. Considering that the scattering matrix **S** depends on the location information of *N* multi-targets, whose position vectors form the matrix **R**:

, (S15)

where is a matrix formed by the unit vector of multi-targets coordinate movements. Denoting translation operator with for the wave momentum of the *i*th target, we can get:

. (S16)

Substituting , for the multi-targets ends up with:

. (S17)

Hence, for arbitrary input information states and output information states , the following equation can be formed:

. (S18)

The above equation shows that the core of multi-target information states acquisition lies in the extraction of the system scattering matrix and the wave momentum of the targets. Essentially, the former represents global information and the latter represents local information. What’s more, the reason that the Neuroute may seem counterintuitive is its abandonment of the local information, which will be discussed briefly in the next section.

***7. A brief explanation of why the Neuroute is non-invasive***

With the physical informed layer (shown in Fig. 2B) in the istGAN8, the Neuroute can generated non-invasively based on the construction shown in Supplementary note S4.

For the Neuroute, it essentially constructs the mapping relationship between the system scattering matrix **S** and the on-demand wave field showing in Fig. S6:

, (S19)

where *f* and *g* are nonlinear mapping relationships fitted by the neural network. For simplification, we approximate *S* to different subcarriers and take it into Fourier series form. Hence, for each scattering matrix element with the angle of arrival and delay inside the scattering channel, the real and imaginary parts can be written as:

, (S20)

, (S21)

here we denote the amplitude coefficient , where is only related to the spatial features of electromagnetic material. Denoting , then the gradient of the scattering matrix relative to the spaces can be written as:

, (S22)

. (S23)

These mathematical expressions exhibit inherent characteristics that are instrumental in designing the network representing them. Firstly, in prior research, neural networks were limited to handling real numbers, necessitating the initial division of the original complex scattering matrix into real and imaginary parts, subsequently concatenated into a real matrix for input into the neural network. However, this process significantly disrupts the correlation within the input data. In contrast, within the gradient formula of the scattering matrix S for space, the expression to the right of the equal sign encompasses the weighted and direct components of the real and imaginary parts of the channel response, respectively. Consequently, istGAN’s partitioning of the original complex channel into real and imaginary parts does not compromise the intrinsic correlation of the data, but rather facilitates the network's assimilation of such non-intrusive data.

Secondly, the original scattering matrix constitutes high-dimensional complex data, posing challenges for processing by neural networks. However, in the Neuroute scenario, the pivotal parameter is the characteristic mean free path *l*. In addition to the existing network's capacity to extract high-dimensional scattering matrix data as output, istGAN also learns straightforward mappings from high-dimensional complex data to low-dimensional simple data, thereby emphasizing the reduction of unnecessary attention overhead.

It is particularly noteworthy that the acquisition time of the scattering matrix typically holds symbolic significance in practical scenarios. Consequently, during the sampling period of the scattering matrix sequence for a propagation path, the symbolic properties undergo minimal changes due to dynamic variations. This ensures that the index of the matrix elements corresponding to the specific propagation path remains fixed during the dynamic alteration of the scattering matrix, thereby enabling the istGAN to assimilate local spatial gradient information without intrusion.

***8. Validation of the Neuroute-based minimized information states***

The main text results show the most commonly used optimal information states acquisition scenario. The actual use of information states acquisition is on demand. For example, in the anti-eavesdropping scenario, we need to carry out adaptive shielding of the target. As a result, we also used the Neuroute for on-demand information states acquisition validation shown in Fig. S7. In this case, we collect data from 13500 sets of scattered scenes, and suppress the information states at the target with the Neuroute adaptively, achieving the effect of non-invasive self-adaptive anti-transmission.

***9. Mathematical model of the parameters estimation precision limit***

To illustrate our approach to judging the information states acquisition effect in the Neuroute, we apply some mathematical tools. Considering the electric field distribution related to the unknown parameter and sample points around the *m*th target with *N* multi-targets totally, with representing the additive Poisson noise caused by our measurements. The Fisher information is generally expressed by9:

. (S24)

For multi-targets, is the information states carrier obtained after environmental transformation of wave field sent by the Neuroute generator following , So the elements on the diagonal of evolve to:

(S25)

where can be considered as mode factors contributing to the test point, generating:

(S26)

Considering unitary *S* and substituting , we can obtain:

(S27)

Therefore, the operator *Q* can be conveniently used to represent the effect of multi-targets information states acquisition, which is also the original intention of choosing it as the Neuroute’s proof-of-concept verification. However, using the Fisher information matrix as the objective function to solve the optimal information states often requires repeated iterations. As shown in Fig. S8, information states optimization using heuristic methods can achieve a convergence effect similar to the Neuroute, but extremely time-consuming due to the iterations. The Neuroute shares the same principle with traditional methods and can achieve efficient and rapid adaptive effects.


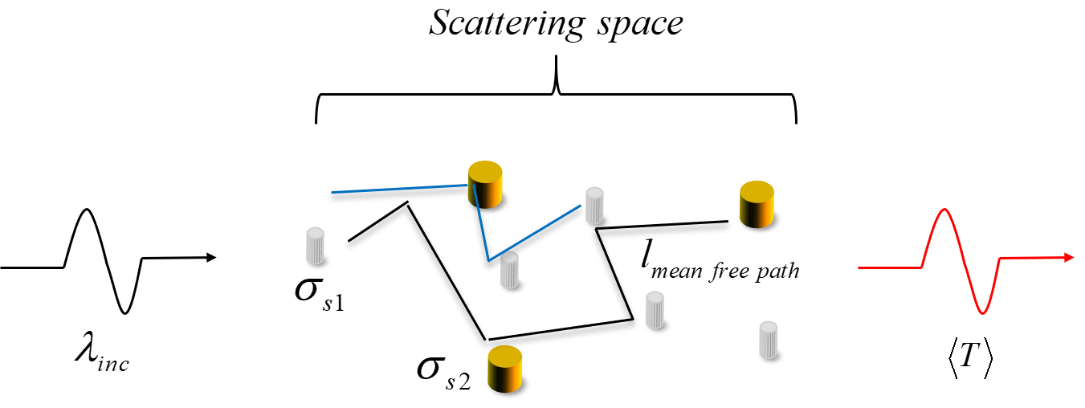


Fig. S1 Schematic diagram of physical model of scattering environment. The incident wave field scatters many times in constructed scattering spaces, the black line and the blue line respectively represent different scattering paths. Finally, the mean free path *l* is obtained statistically, calculated using scattering cross section or measured using transmission rate .


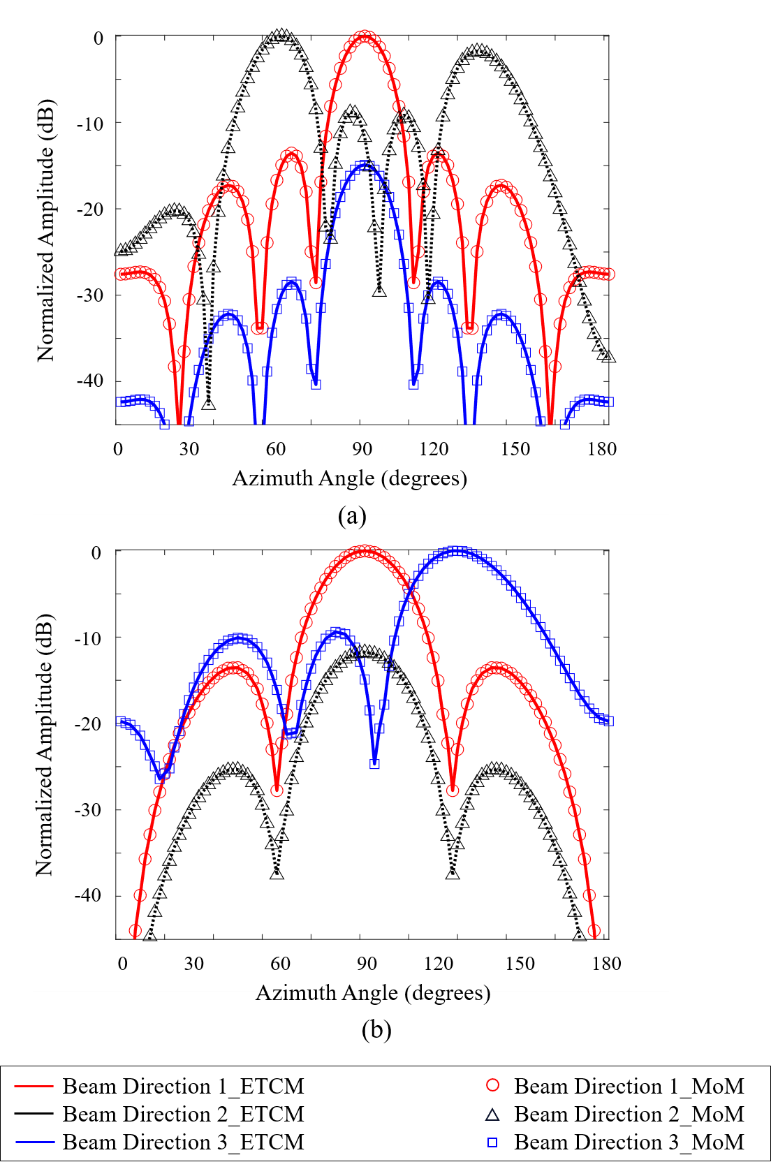


Fig. S2 Comparison results for coupling ports between our element theory of characteristic mode (ETCM) method and traditional full-wave method. The figure shows the normalized radiation pattern when the number of ports is 8, and the numerical comparison is made with the commercial software FEKO and the traditional algorithm moment method (MoM).


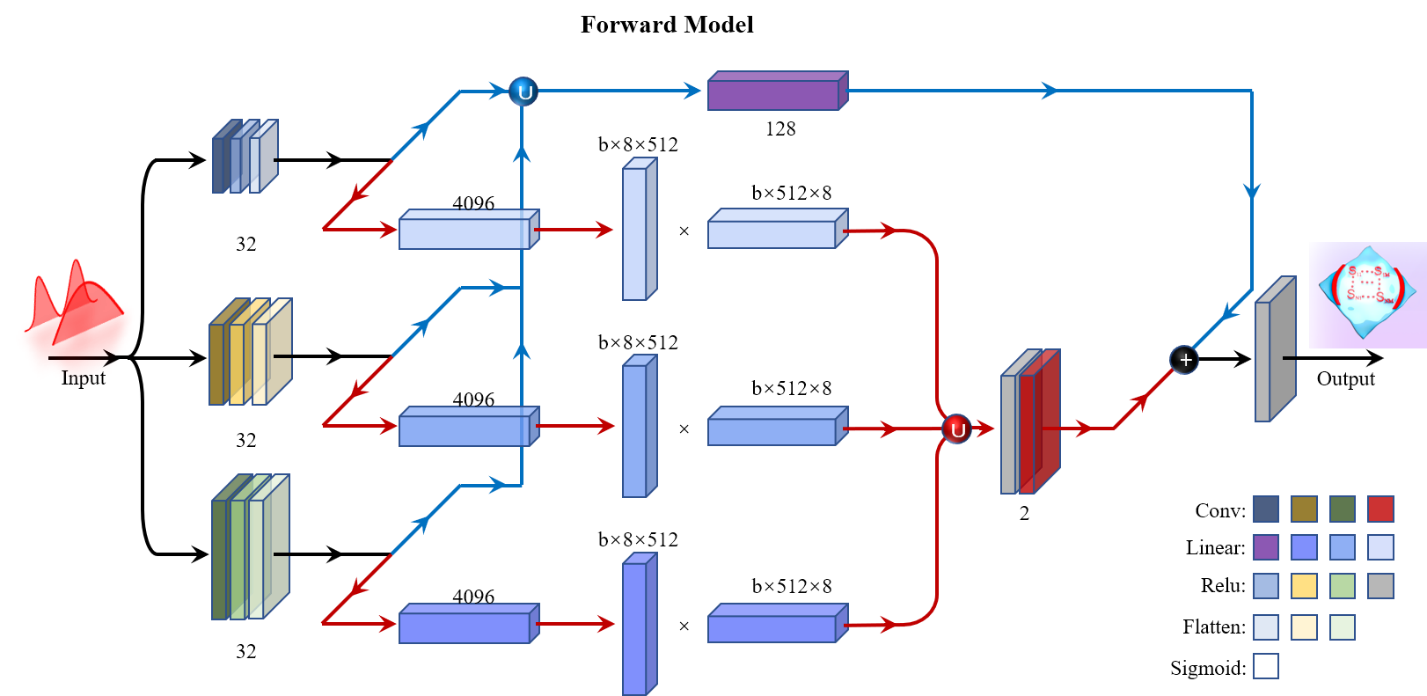


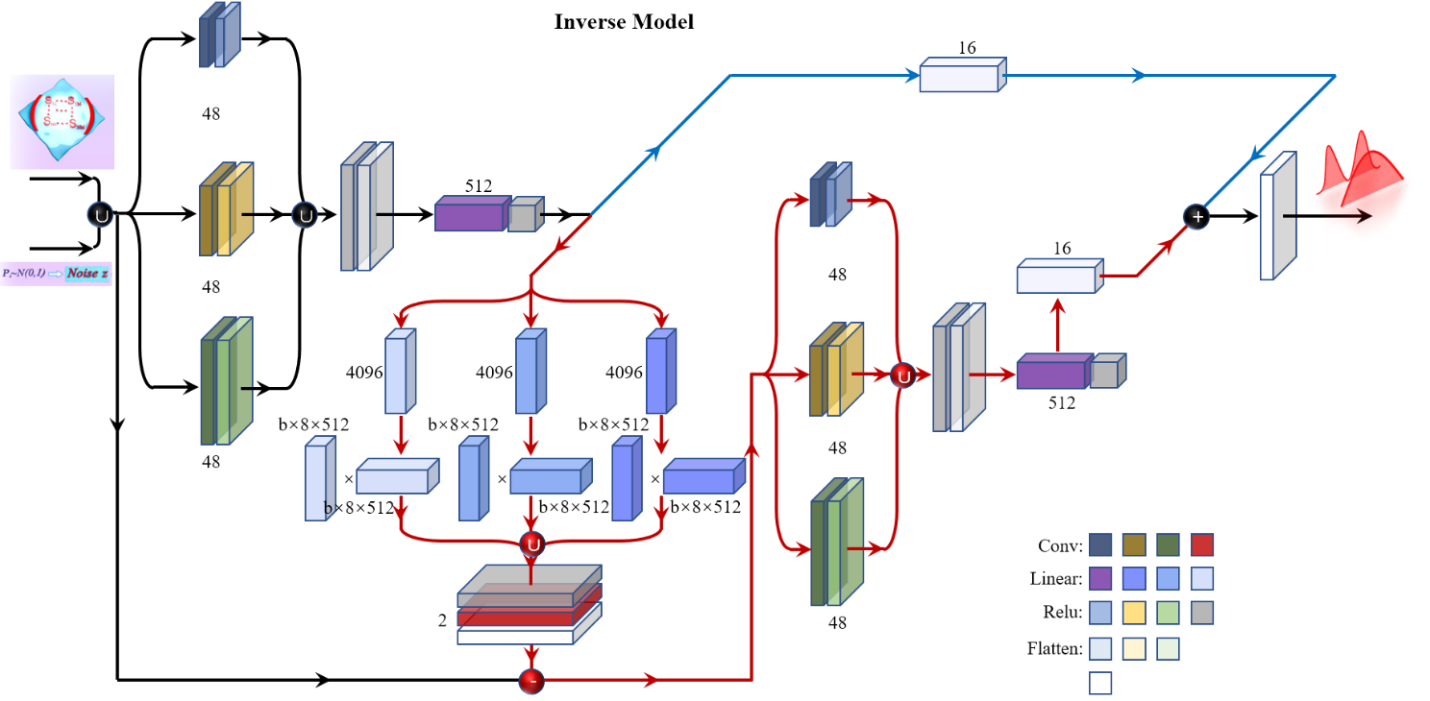


Fig. S3 Architecture of the istGAN.


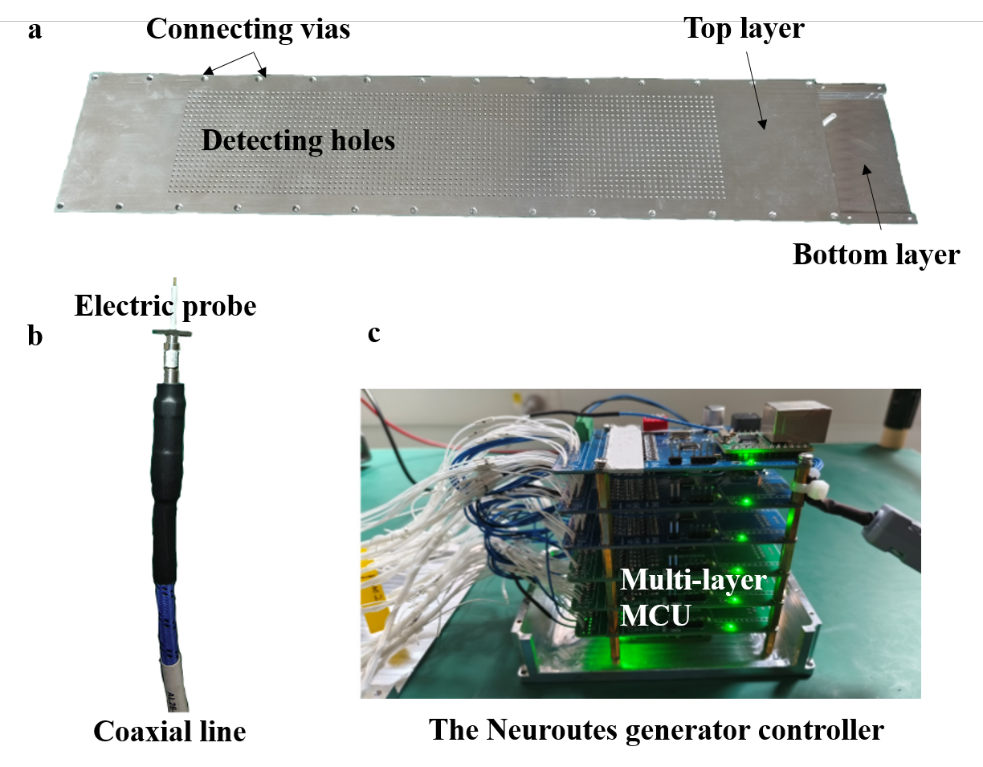


Fig. S4 Experimental configuration. (a) A modified rectangular waveguide is used as the basis for scattering space equivalence. To detect the field inside the waveguide, we made 5mm*5mm detection holes on the top layer. The top layer and the bottom layer are fixed by connecting holes, which are convenient to disassemble, facilitating the placement of scatterers inside the waveguide. (b) The electric field probe is connected with the coaxial line, placed into the detecting holes for acquiring the electric field intensity, and the other end is connected with the VNA (Ceyear 3672D). (c) The multilayer microprogrammed control unit (MCU) mainly responsible for two tasks, one is to receive the data processed by the upper computer (optionally, the embedded istGAN for intelligent analysis), and the other is to issue phase shift (TLDA-8G12G-30-6) and attenuation (TLDP-8G12G-360-6) instructions to the connected Neuroute generator.


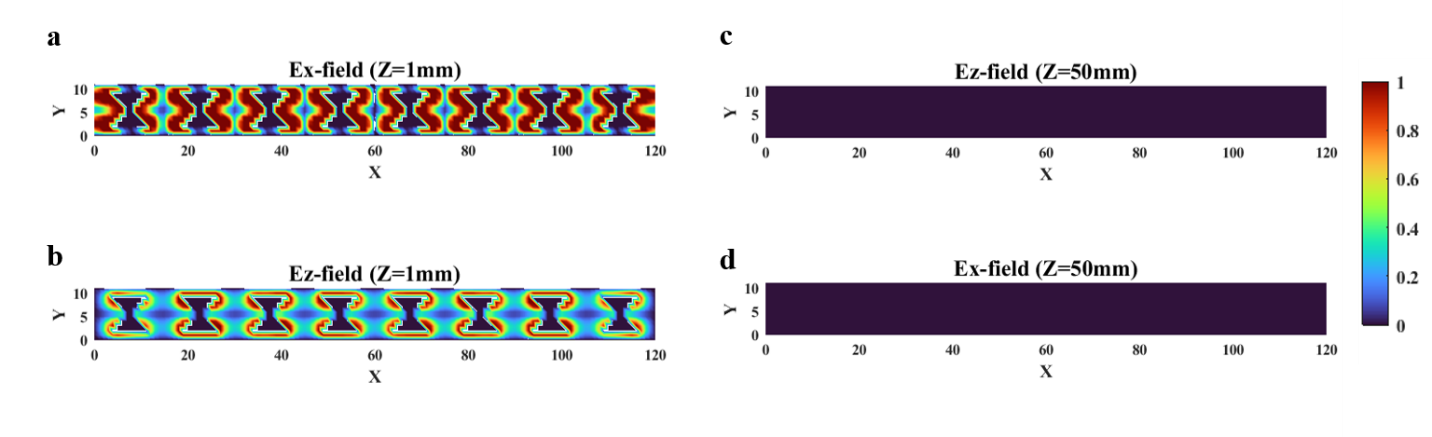


**Fig. S5** **Near-field components verification in experimental model.** Calculated electric field components in X and Z directions (The Y direction is the direction of the transverse electrical mode that provides the main mode). (a)(b) show the distribution of two electric field components for Z=1mm (The Z direction is the propagation direction of the Neuroute), while (c)(d) Z=50mm. It can be observed that the scatterer configured at a certain distance can avoid the influence of the near field components of the radiation sources.


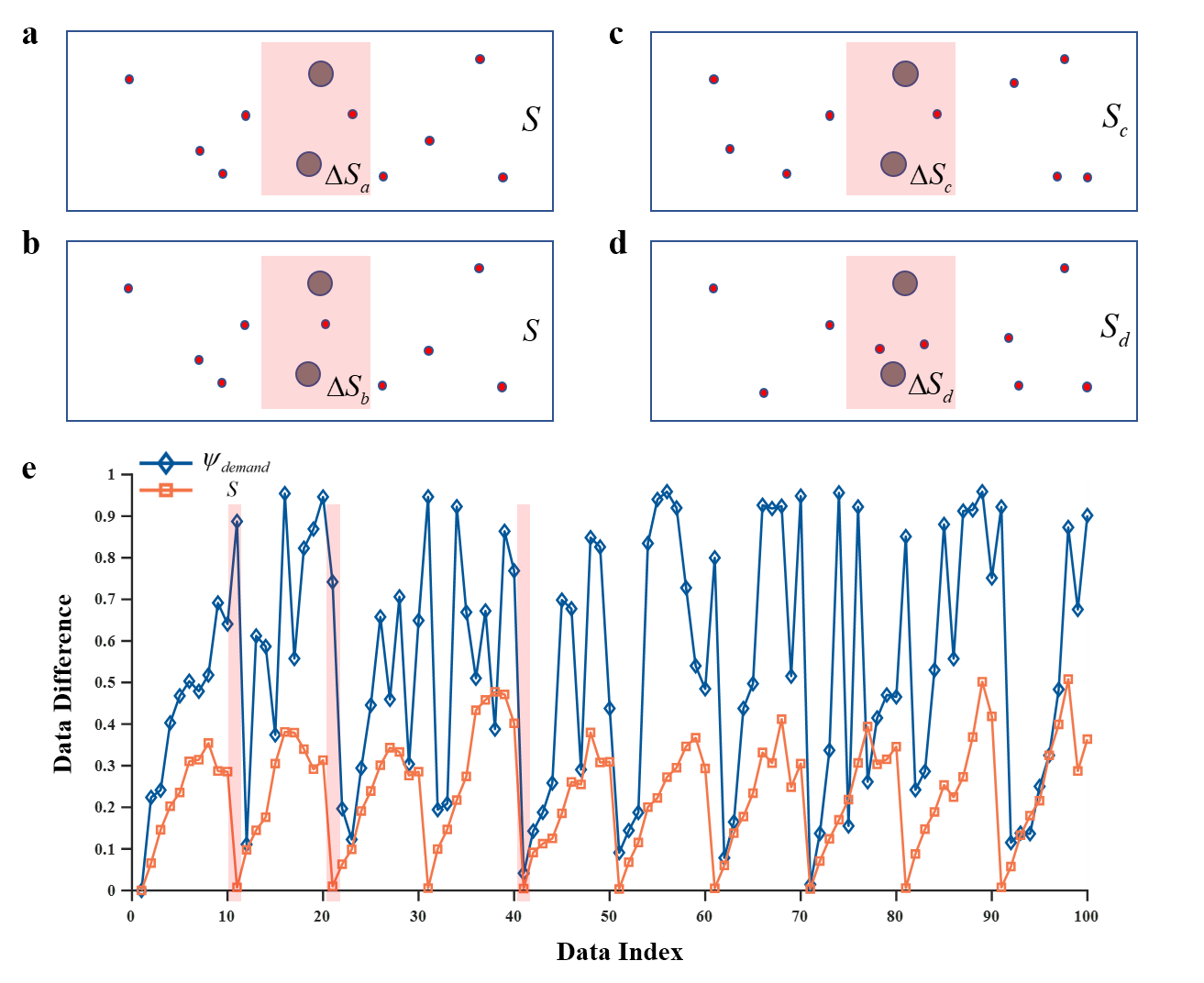


**Fig. S6 Mapping problems between global information and local information for the Neuroute.** (a) and (b) reveal that the same *S* might correspond to different . The perturbations near multi-targets will correspond to different local information and , but the measured global information *S* is the same, eventually corresponding to different . (c) and (d) denote that the same might correspond to different **S**. Imagining a very coincidental situation that , where and , corresponding to the same apparently. (e) 100 samples intercepted from the dataset, and the difference is carried out with the first group of data as reference. If the difference is close to 0, it is considered the same. Several sets of data presented in the red box clearly show the same *S* might correspond to different . In addition, due to the extremely strict conditions, there is no obvious multiple *S* corresponding to the same in our data set, so it can be regarded as a one-to-many problem rather than a many-to-many problem in the design of the istGAN.


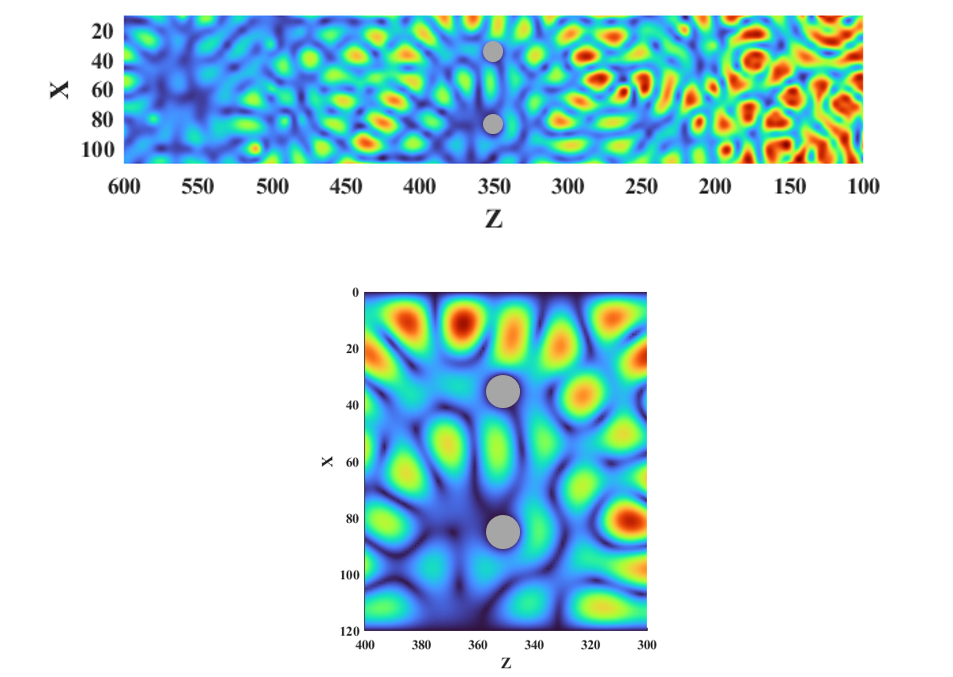


**Fig. S7 Example of anti-transmission using the Neuroute.** By replacing the labels with anti-transmission eigenvectors when training the istGAN, the effect of multi-targets information masking can be rendered after generating the Neuroute.


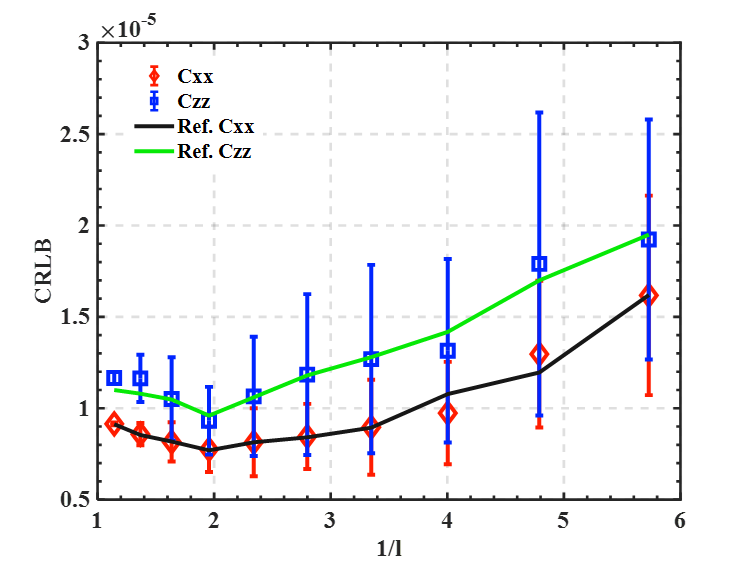


**Fig. S8 Comparation between the traditional method and the Neuroute.** The ability of information states acquisition of the Neuroute is verified by a heuristic algorithm (the genetic algorithm). Because the convergence value of the heuristic algorithm is not stable, we use the best value after ten optimizations, and also use the CRLB as the objective function for comparison. Clearly, the Neuroute is reaching the parameters estimation precision limits without iterations.

**Table S1 | Computational cost for the multi-ports Neuroute generator** **(CPU: i7-7700 AT 3.60 GHz, RAM: 16 GB).**


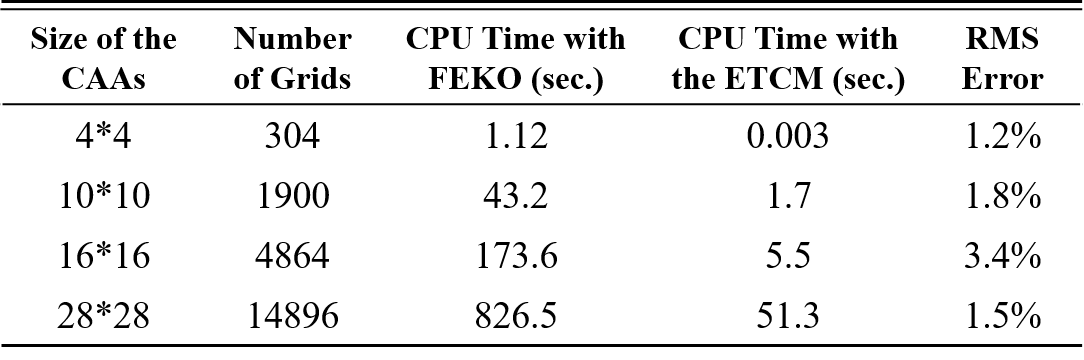


Movie S1 Comparison of the process of generating wave field routes with the classical GWS operator and the process of generating the Neuroute.

**Movie S2 The transmission of video inside dynamic scattering spaces.**

**References**

1. Kelly K. L., Coronado E., Zhao L. L. & Schatz G. C. The Optical Properties of Metal Nanoparticles:  The Influence of Size, Shape, and Dielectric Environment. *The Journal of Physical Chemistry B*. **107**, 668-677 (2003).

2. Lagendijk A. & van Tiggelen B. A. Resonant multiple scattering of light. *Physics Reports*. **270**, 143-215 (1996).

3. Chen Y., Sgrignuoli F., Zhu Y., Shubitidze T. & Dal Negro L. Enhanced wave localization in multifractal scattering media. *Physical review. B*. **107**, (2022).

4. John S. Strong localization of photons in certain disordered dielectric superlattices. *Phys. Rev. Lett.* **58**, 2486-2489 (1987).

5. Li R., Li D., Ma J., Feng Z., Zhang L. & Tan S. et al. An Electromagnetic Information Theory Based Model for Efficient Characterization of MIMO Systems in Complex Space. *IEEE T. Antenn. Propag.* **71**, 3497-3508 (2023).

6. Ghosal S., Sinha R., De A. & Chakrabarty A. Characteristic Mode Analysis of Mutual Coupling. *IEEE T. Antenn. Propag.* **70**, 1008-1019 (2022).

7. Tayli D., Capek M., Akrou L., Losenicky V., Jelinek L. & Gustafsson M. Accurate and Efficient Evaluation of Characteristic Modes. *IEEE T. Antenn. Propag.* **66**, 7066-7075 (2018).

8. Del H. P., Yeo K. B., Besnier P. & Davy M. Coherent Wave Control in Complex Media with Arbitrary Wavefronts. *Phys. Rev. Lett.* **126**, 193903 (2021).

9. Shechtman Y., Sahl S. J., Backer A. S. & Moerner W. E. Optimal point spread function design for 3D imaging. *Phys. Rev. Lett.* **113**, 133902 (2014).
