# Supplementary material for: Non-Invasive Self-Adaptive Information States’ Acquisition inside Dynamic Scattering Spaces
Source: Research (Wash D C). 2024 May 31;7:0375. doi: 10.34133/research.0375 (PMC11140760; doi:10.34133/research.0375)

## Slide 1
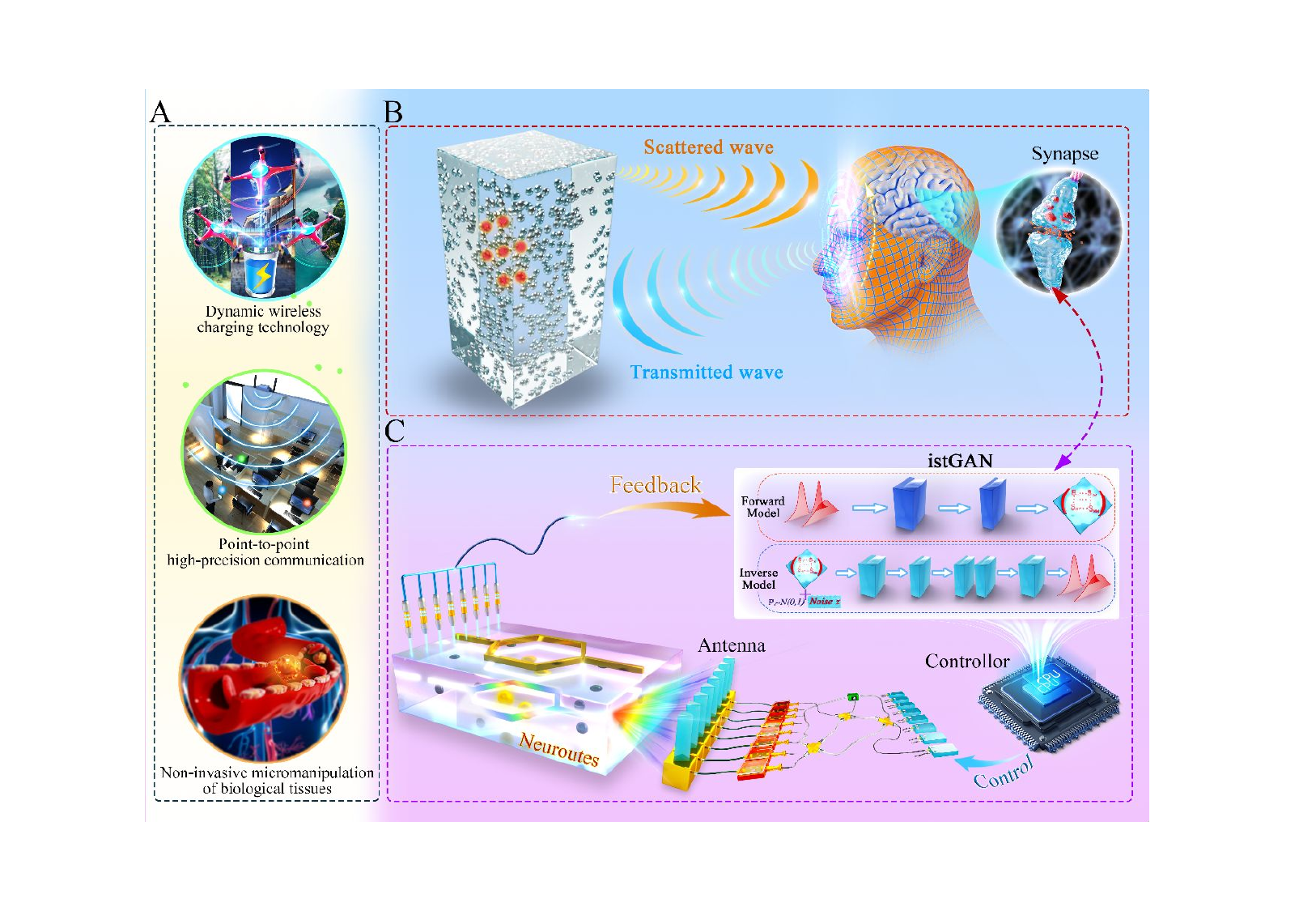

## Slide 2
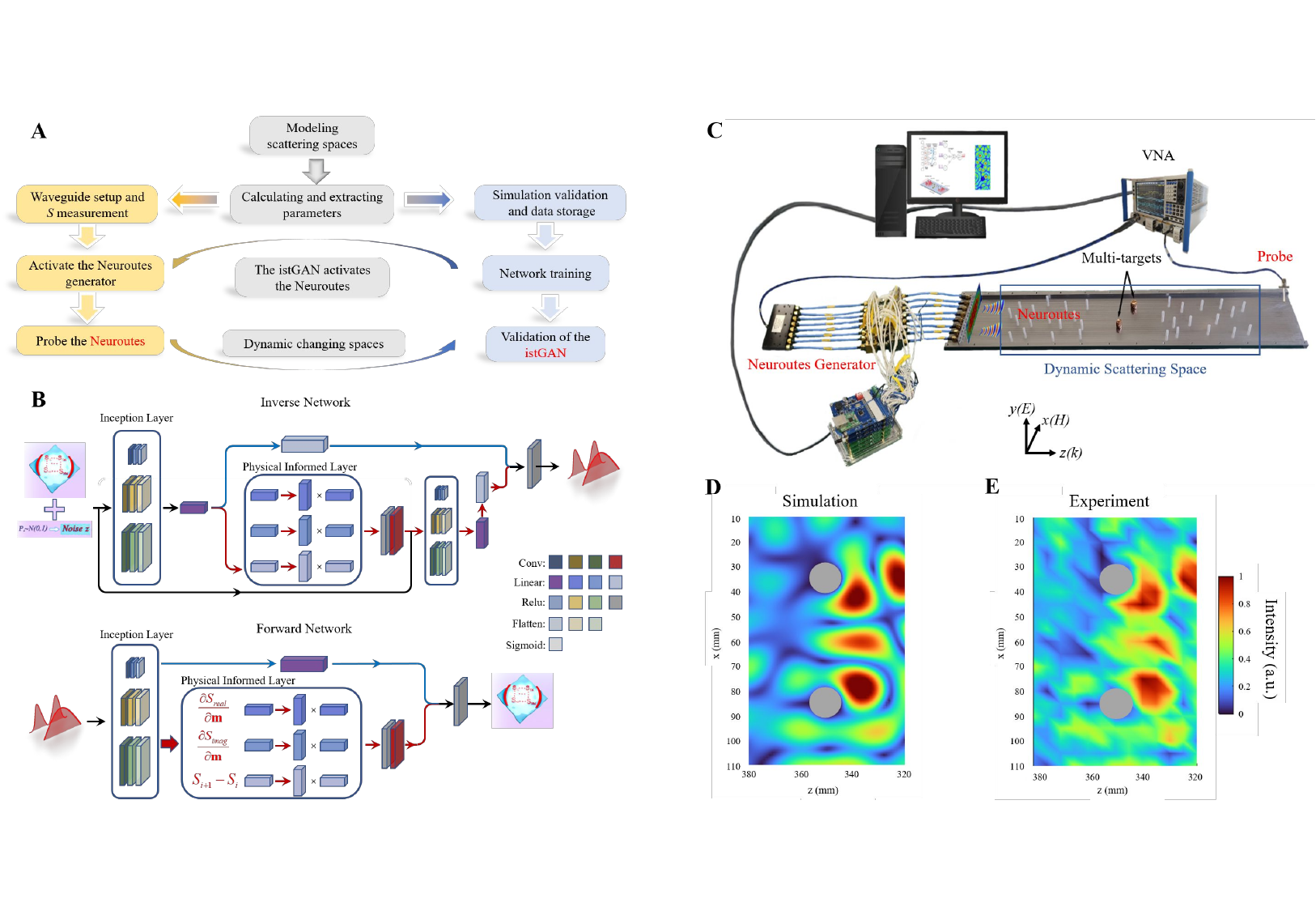

## Slide 3
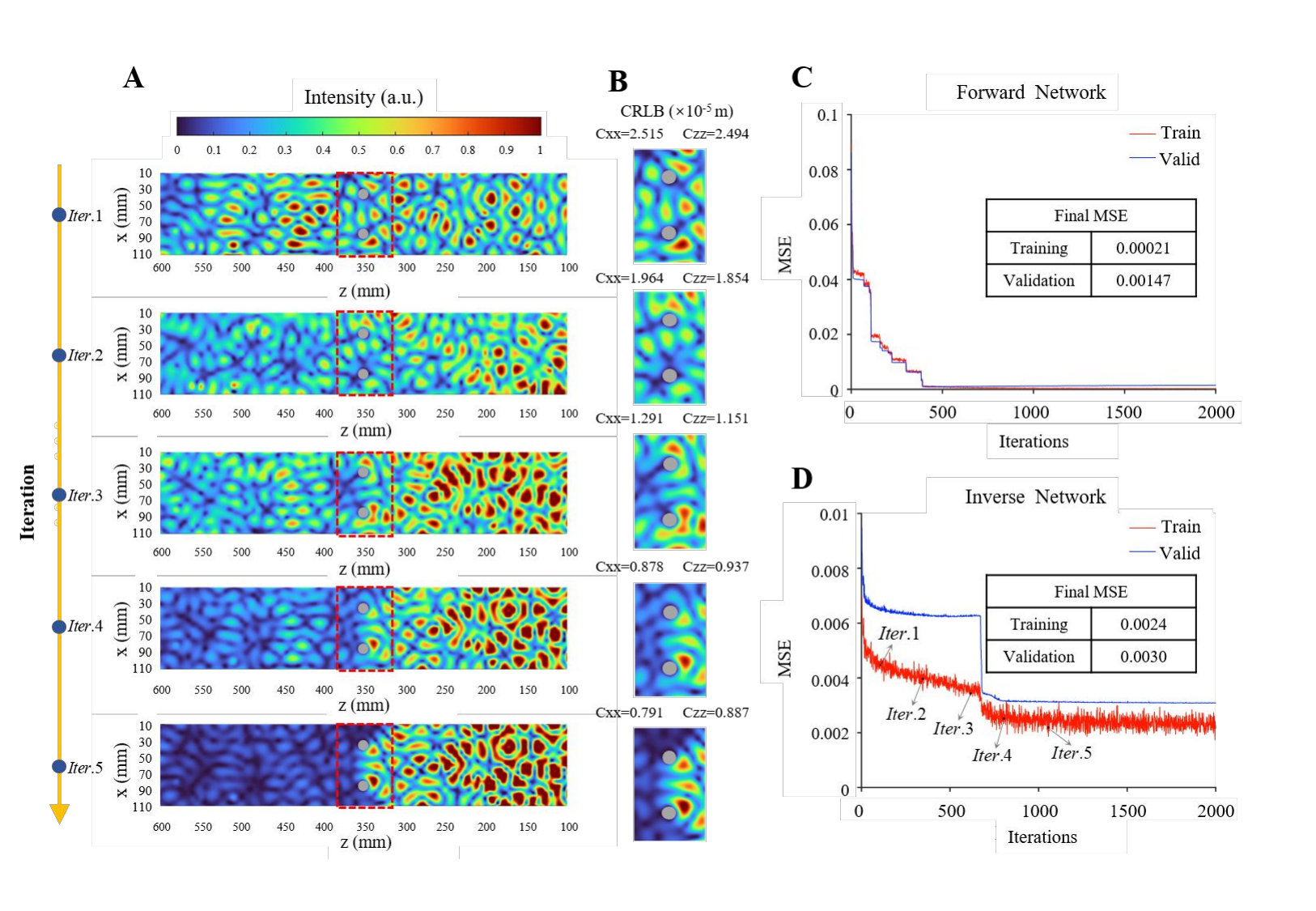

## Slide 4
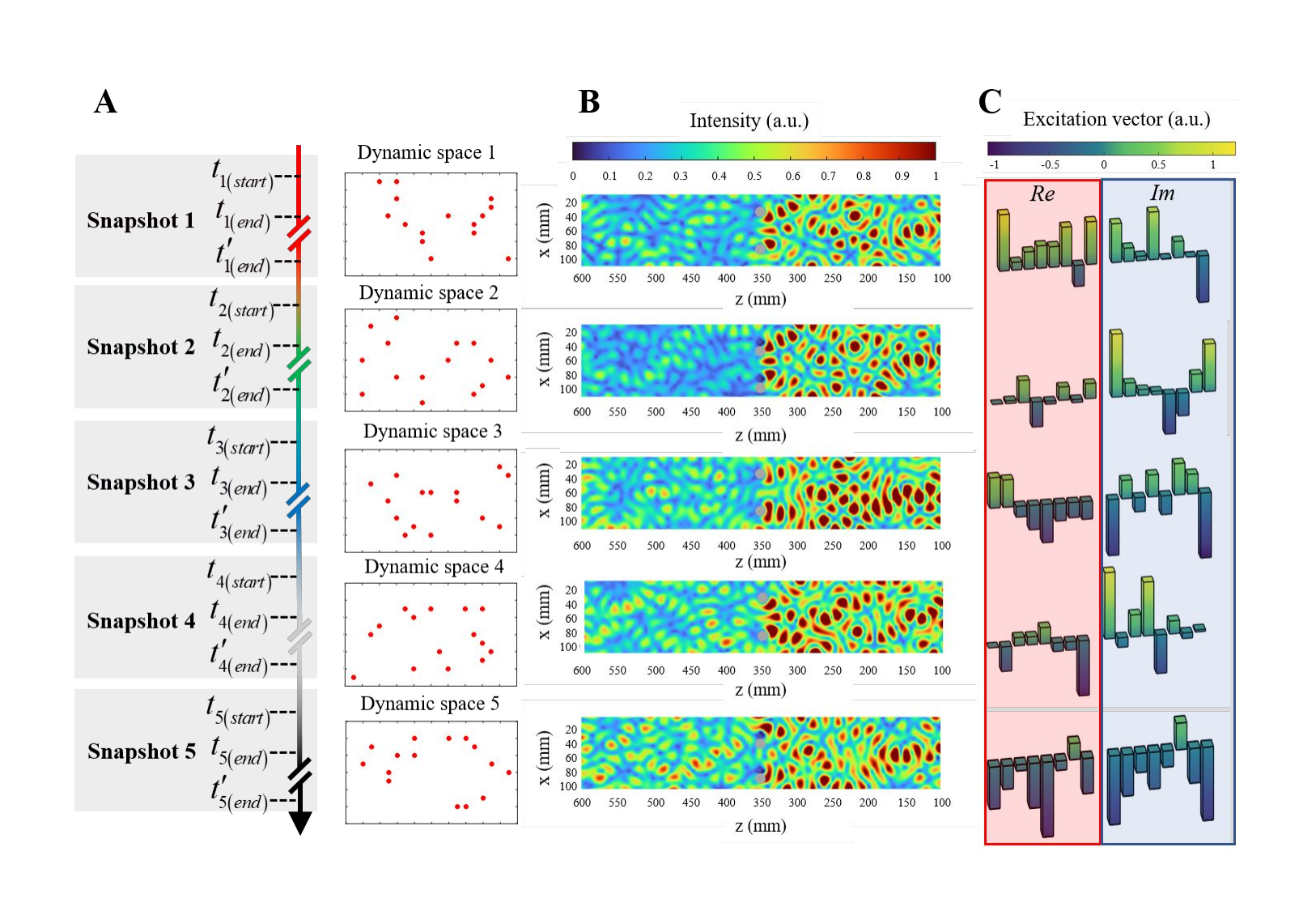

## Slide 5
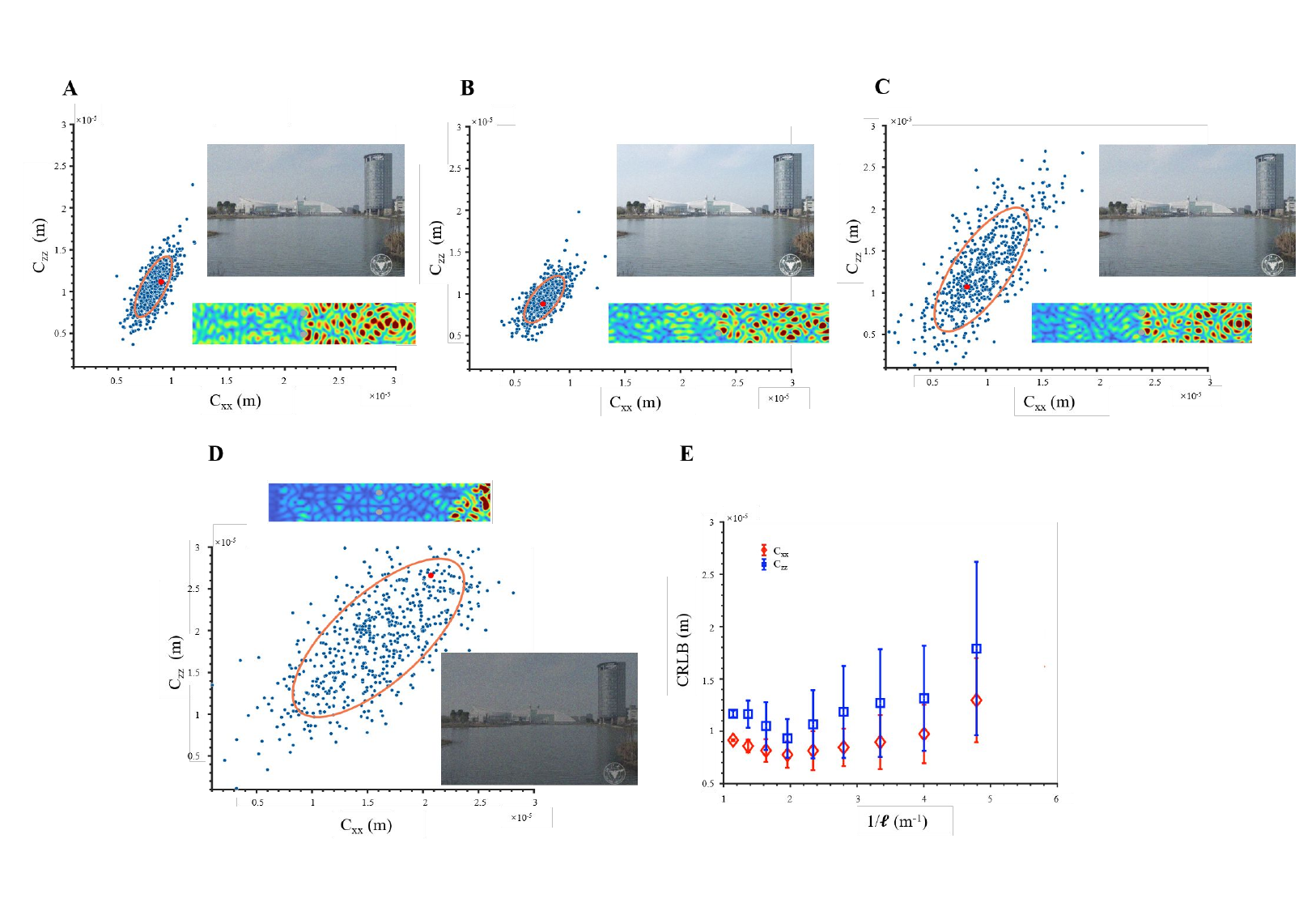

## Slide 6
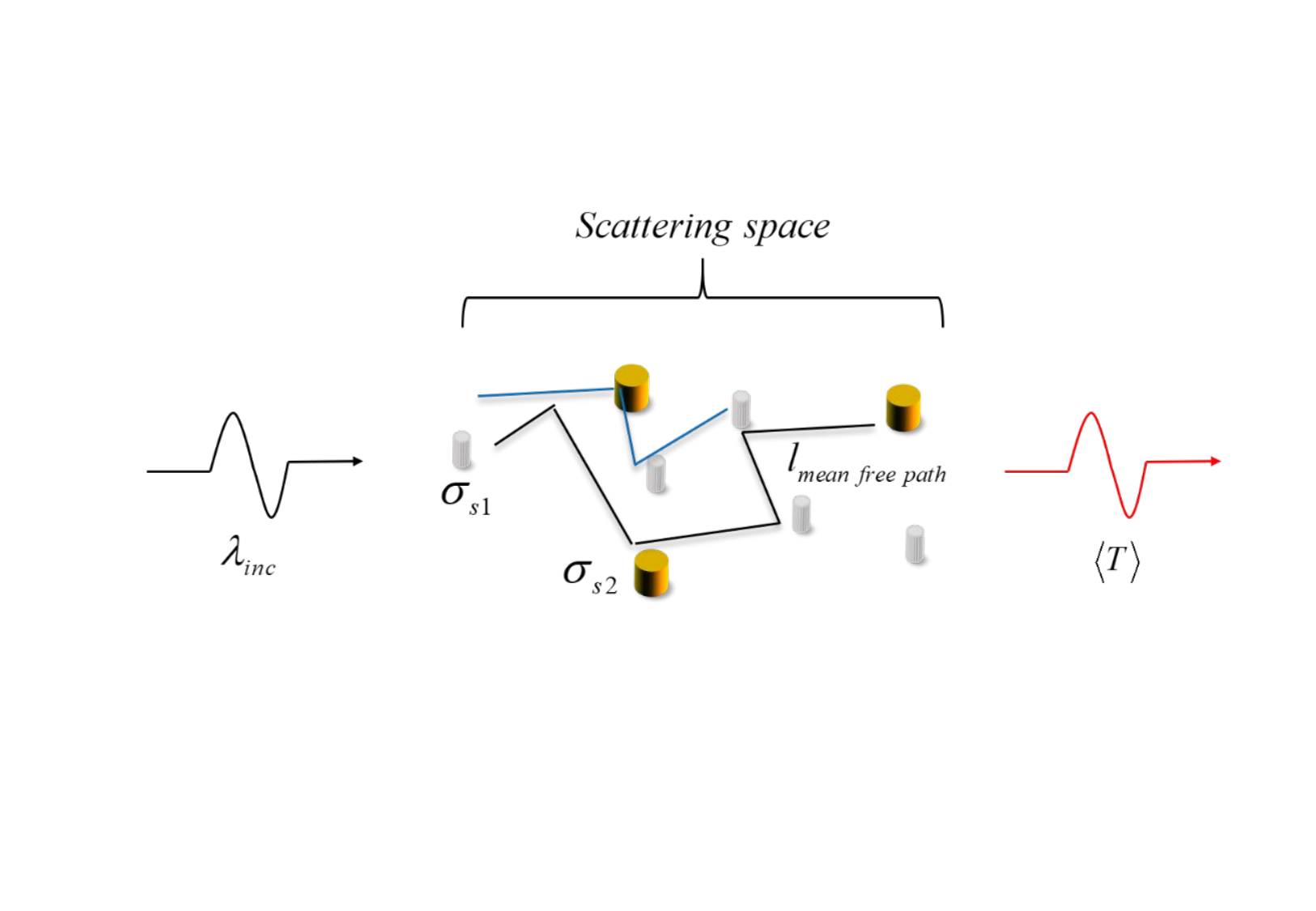

## Slide 7
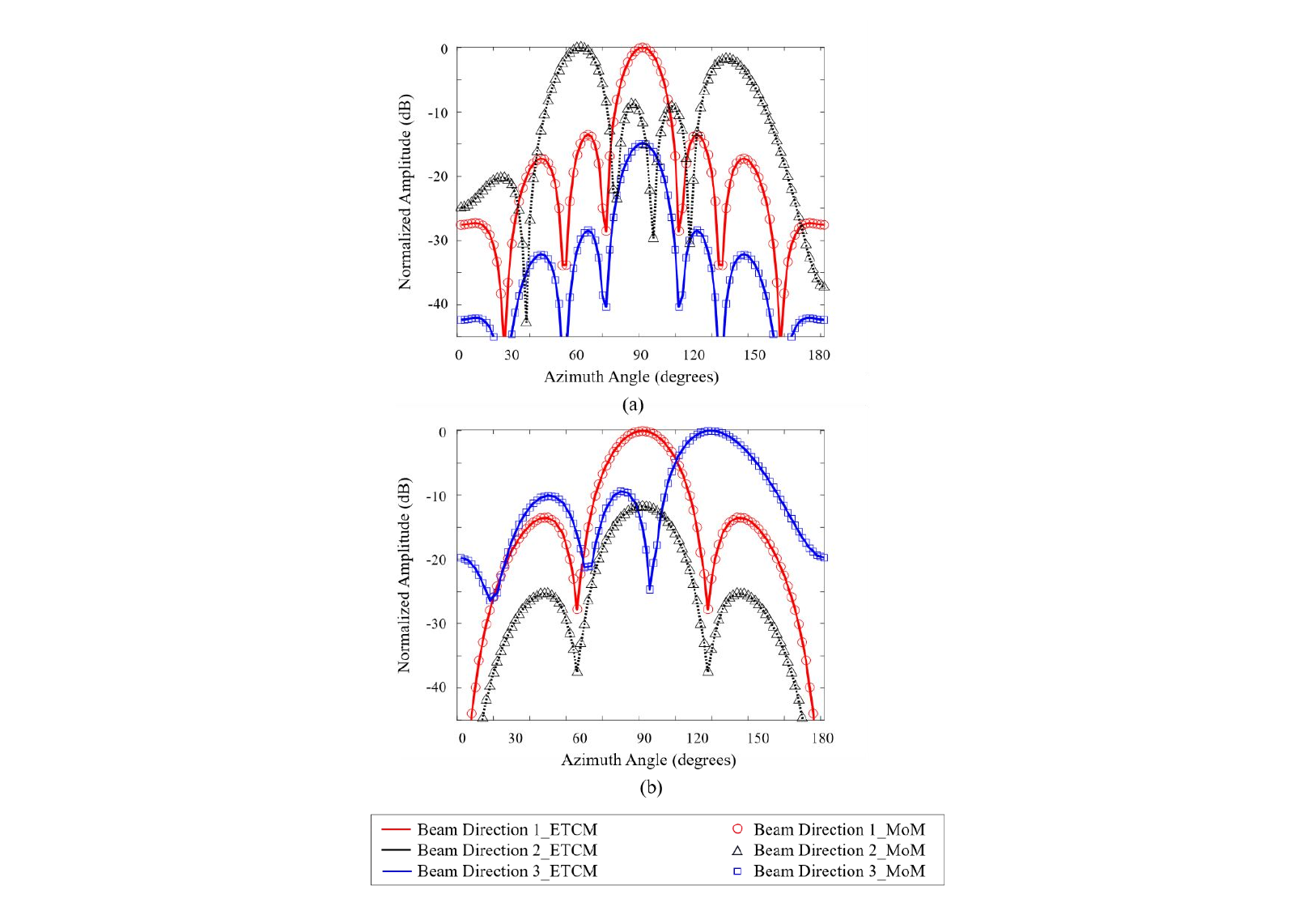

## Slide 8
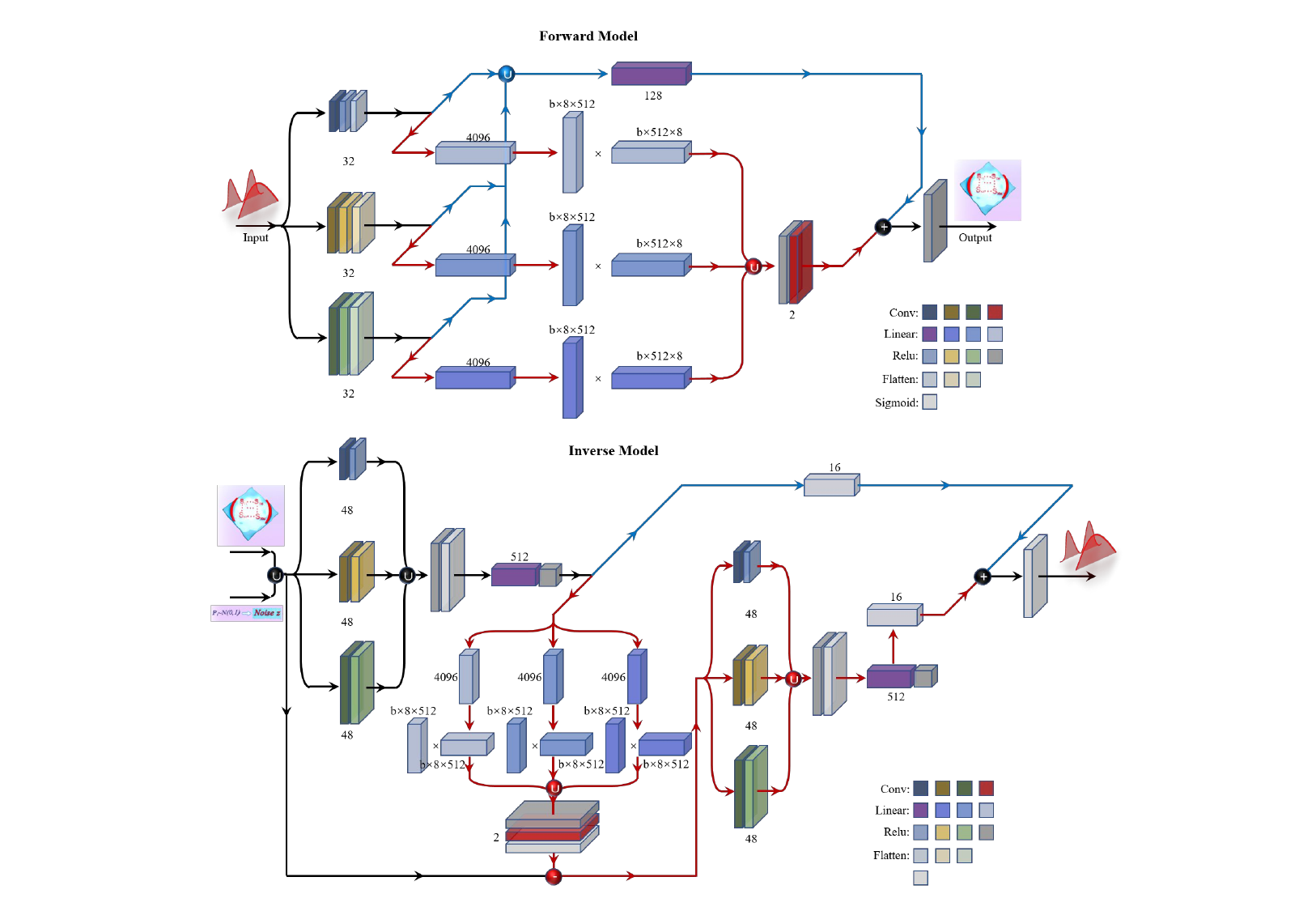

## Slide 9
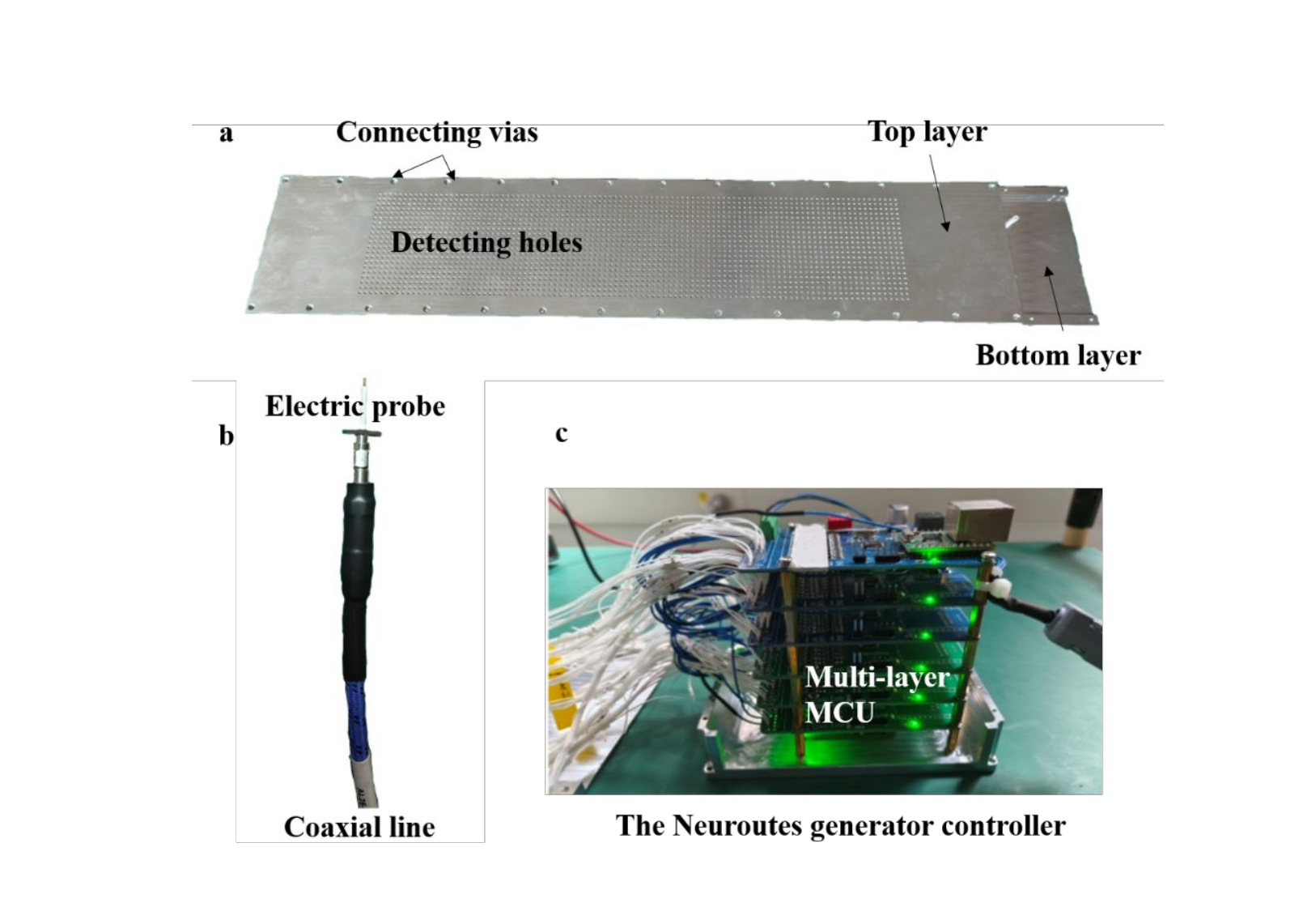

## Slide 10
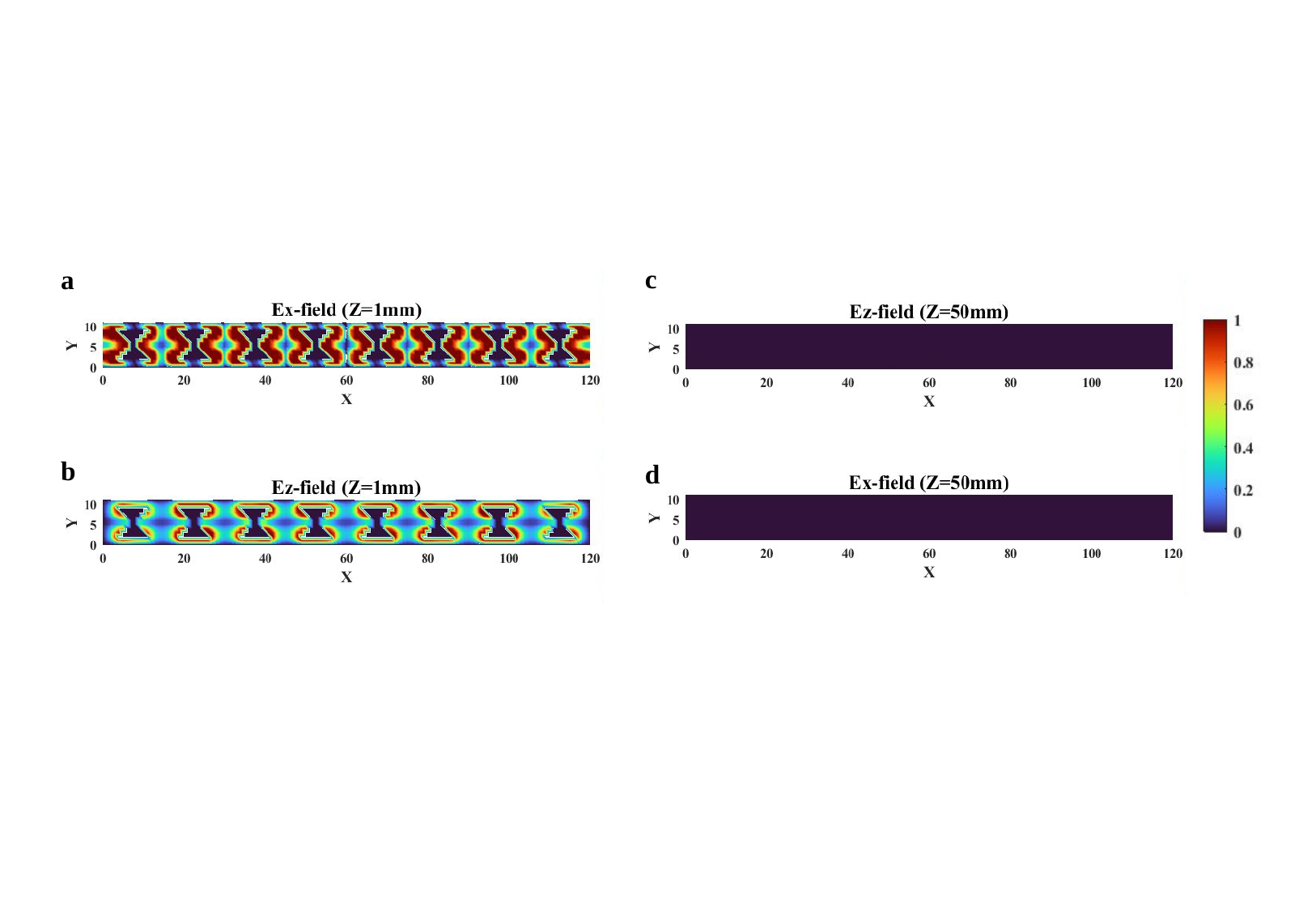

c
a
b
d

## Slide 11
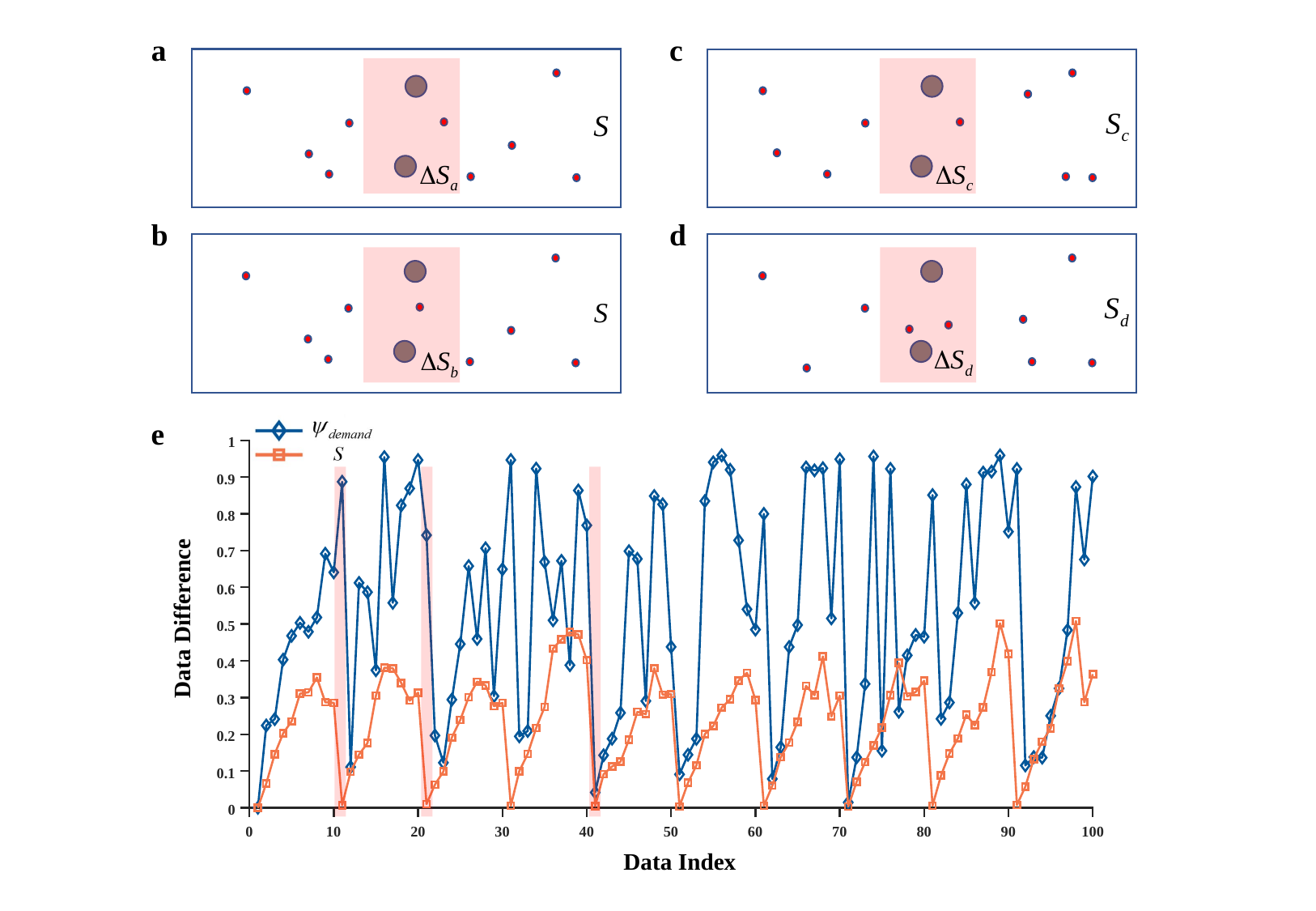

a
c
b
d
Data Difference
Data Index
e

## Slide 12
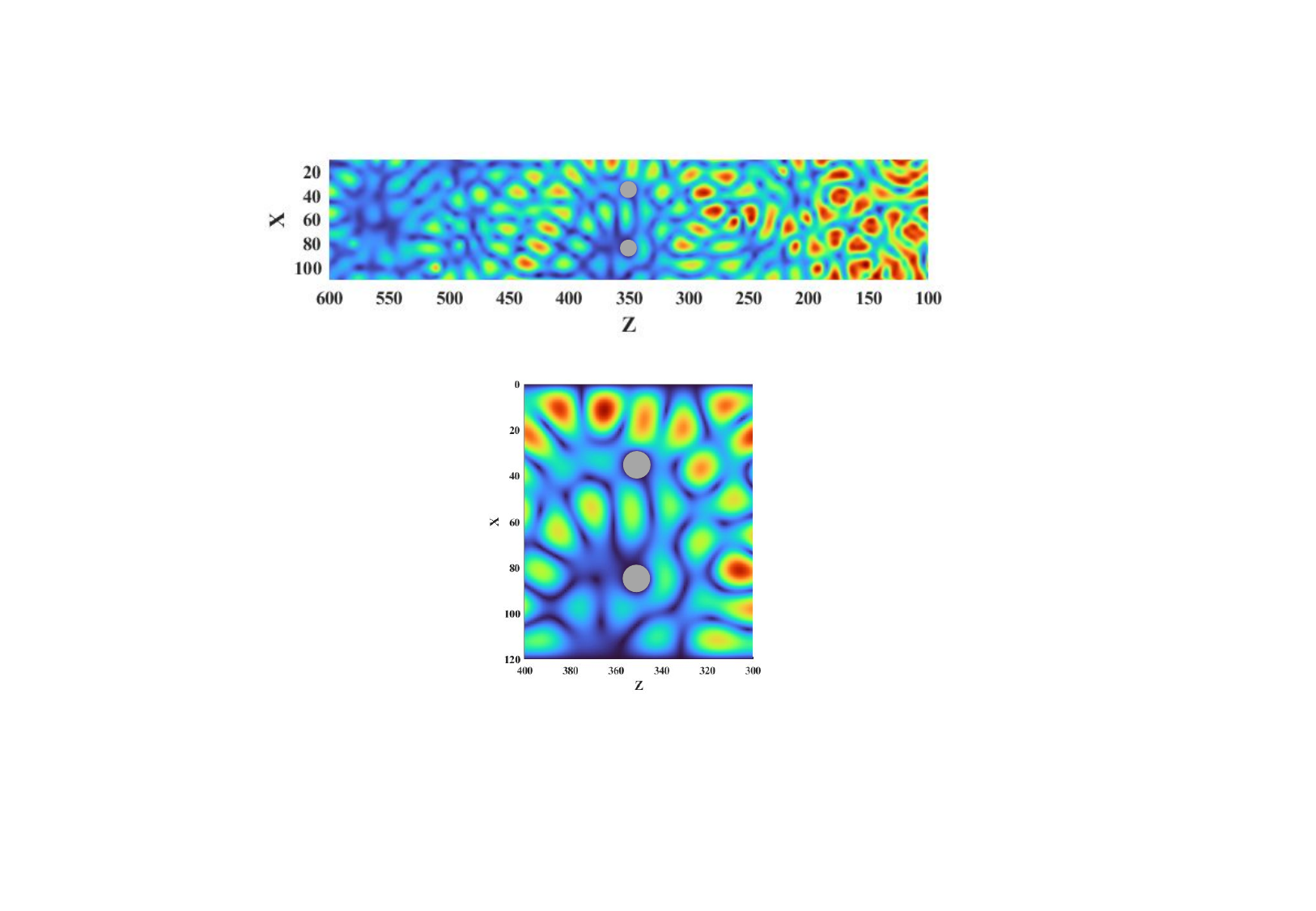

## Slide 13
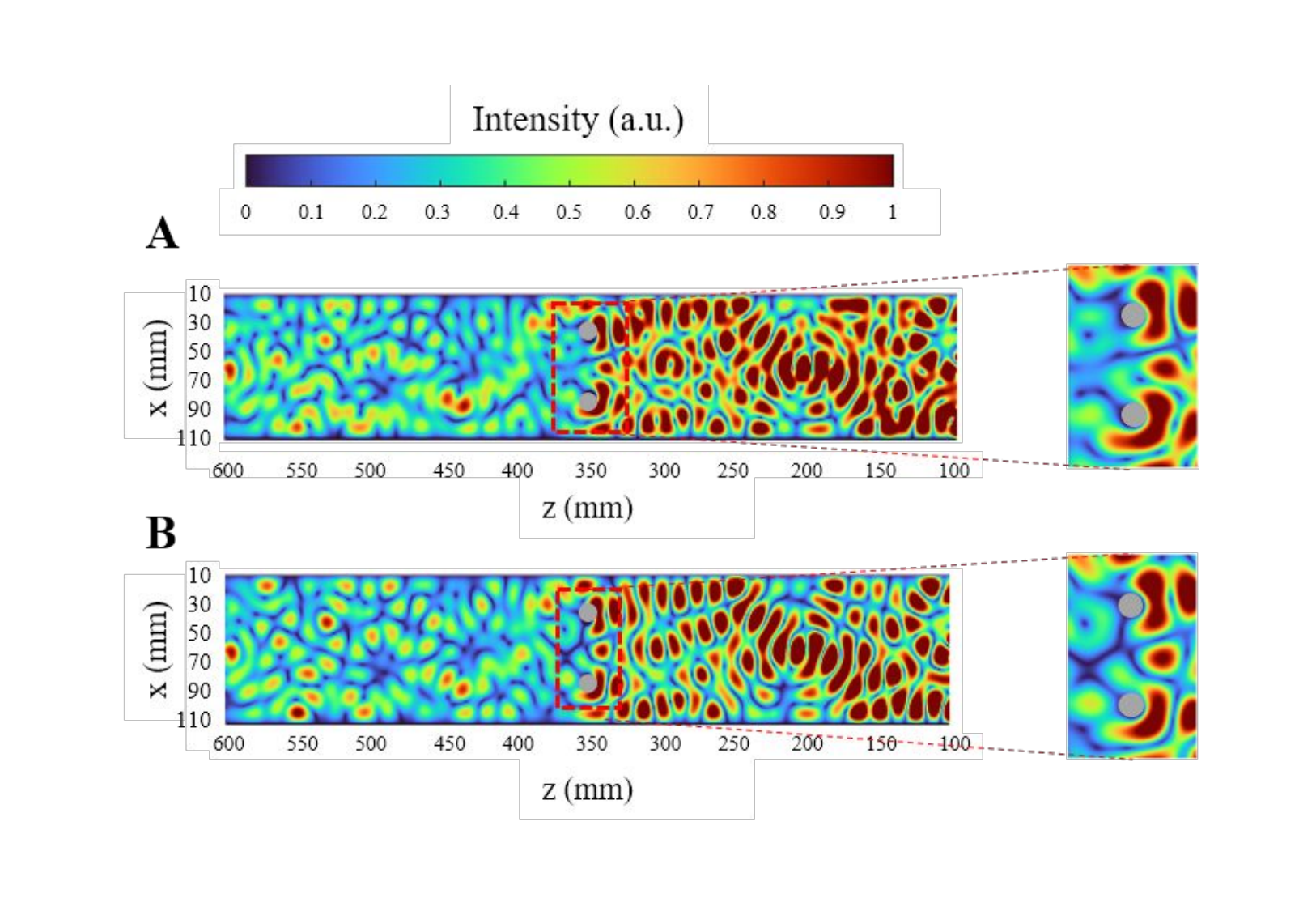

## Slide 14
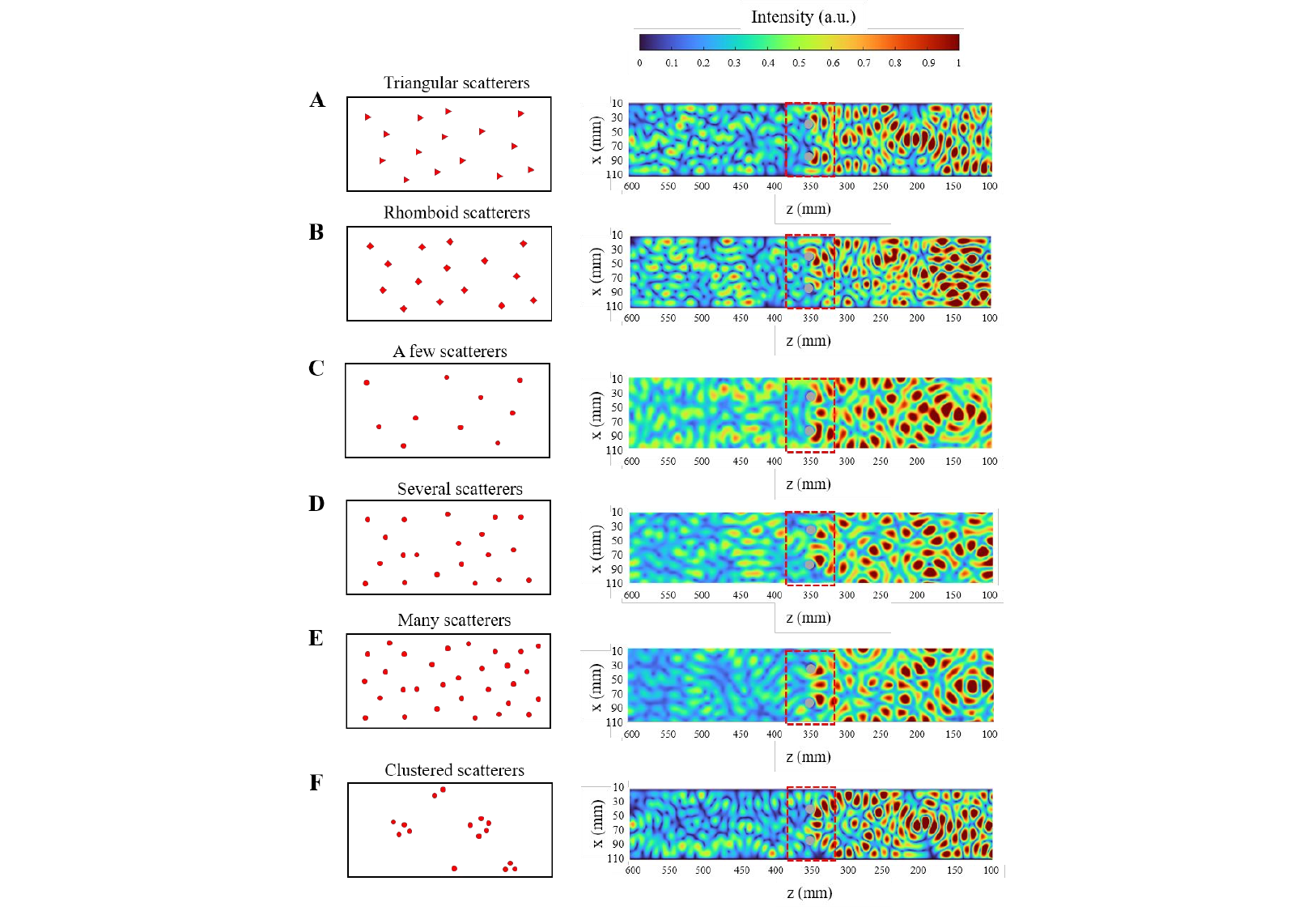

## Slide 15
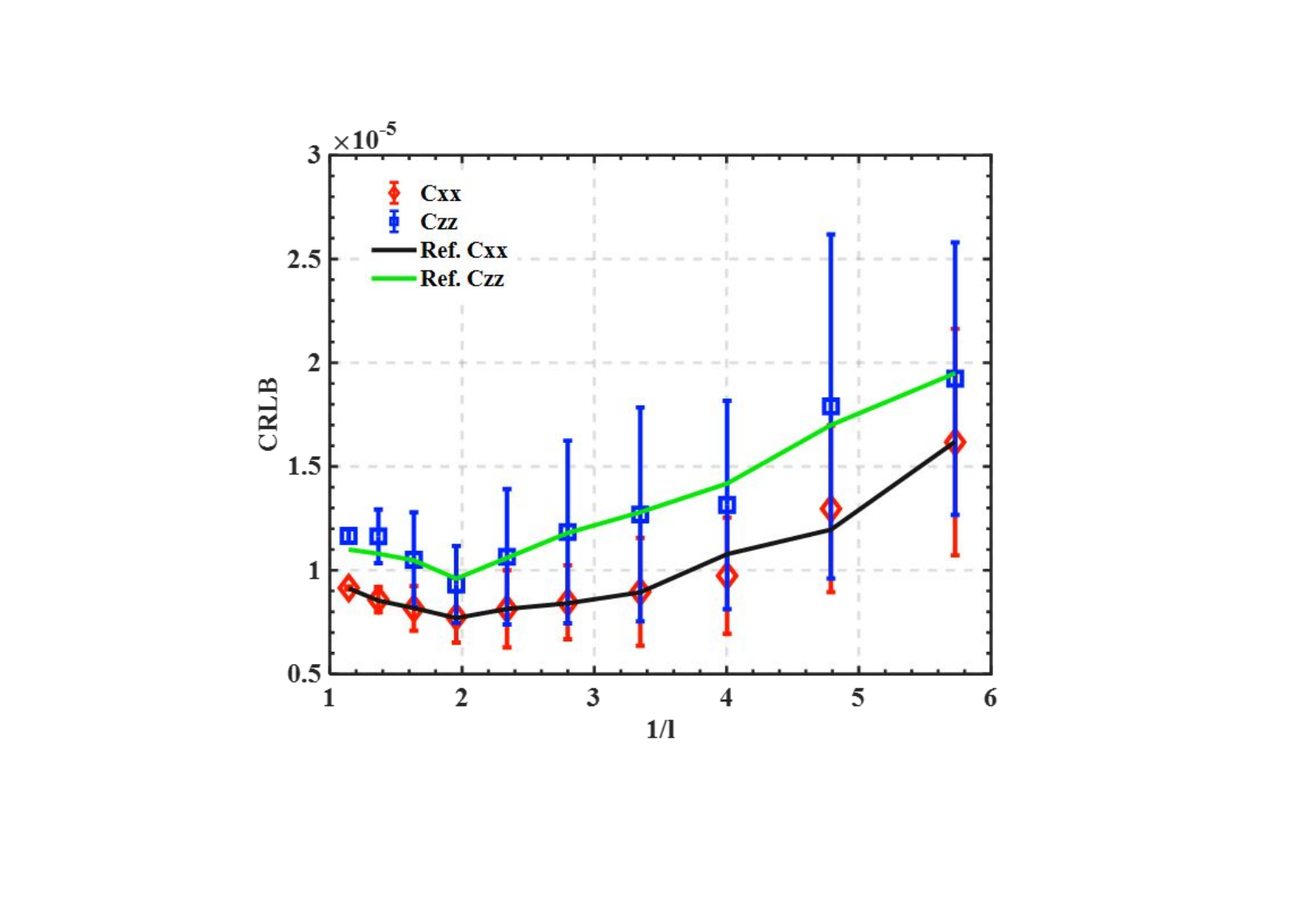

Supplement: Supplementary 1 — Figs. S1 to S8 Table S1 Movies S1 and S2 [file research.0375.f1.zip › picture 4.0.pptx]
