# Supplementary material for: Non-Invasive Self-Adaptive Information States’ Acquisition inside Dynamic Scattering Spaces
Source: Research (Wash D C). 2024 May 31;7:0375. doi: 10.34133/research.0375 (PMC11140760; doi:10.34133/research.0375)
Supplement: Supplementary 1 — Figs. S1 to S8 Table S1 Movies S1 and S2 [file research.0375.f1.zip › picture 4.1.pdf]

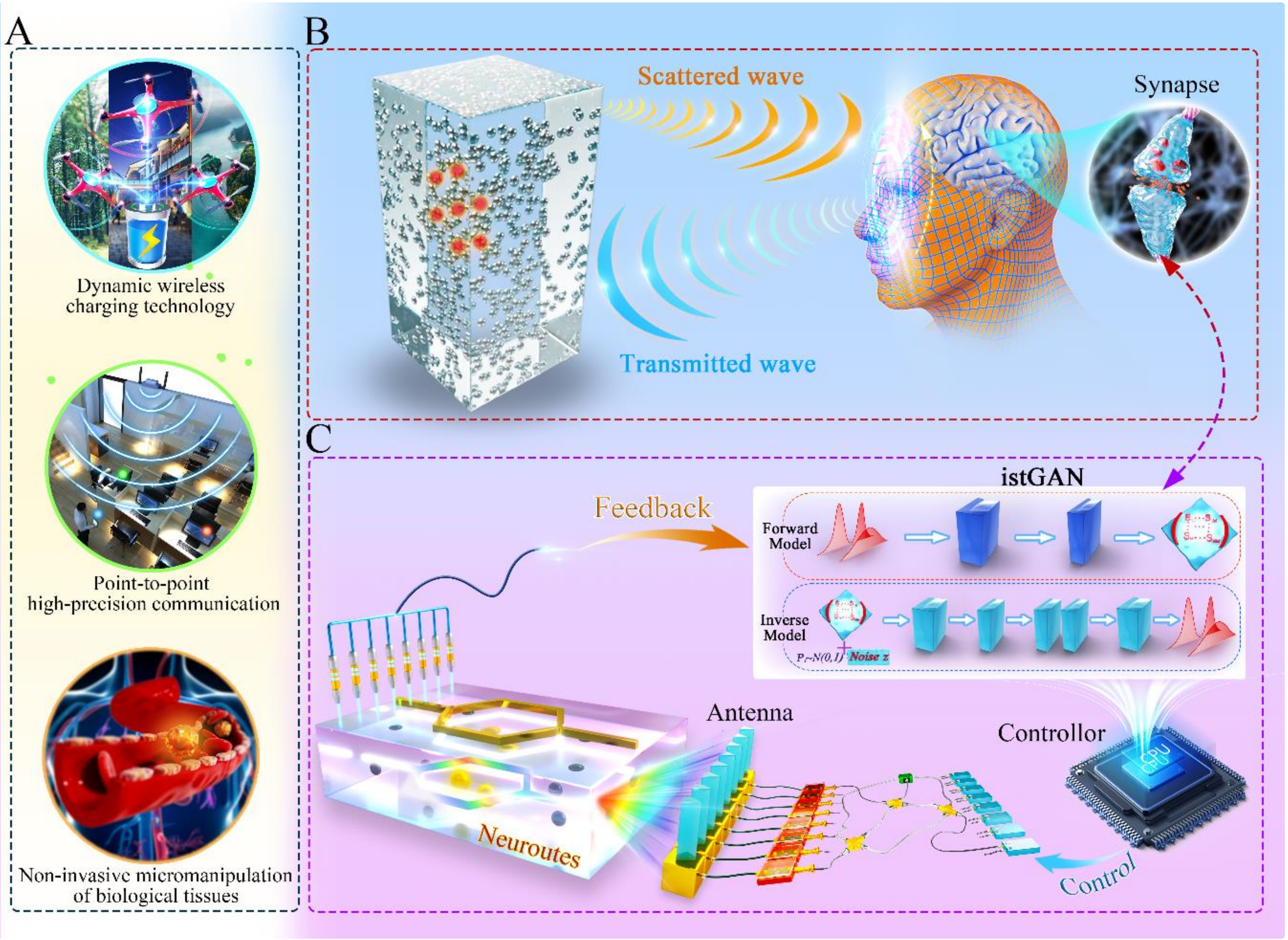
A
Dynamic wireless charging technology
Point-to-point high-precision communication
Non-invasive micromanipulation of biological tissues
B
Scattered wave
Transmitted wave
Synapse
C
istGAN
Feedback
Forward Model
Inverse Model
P~N(0,1) Noise z
Antenna
Controllor
Neuroutes
Control

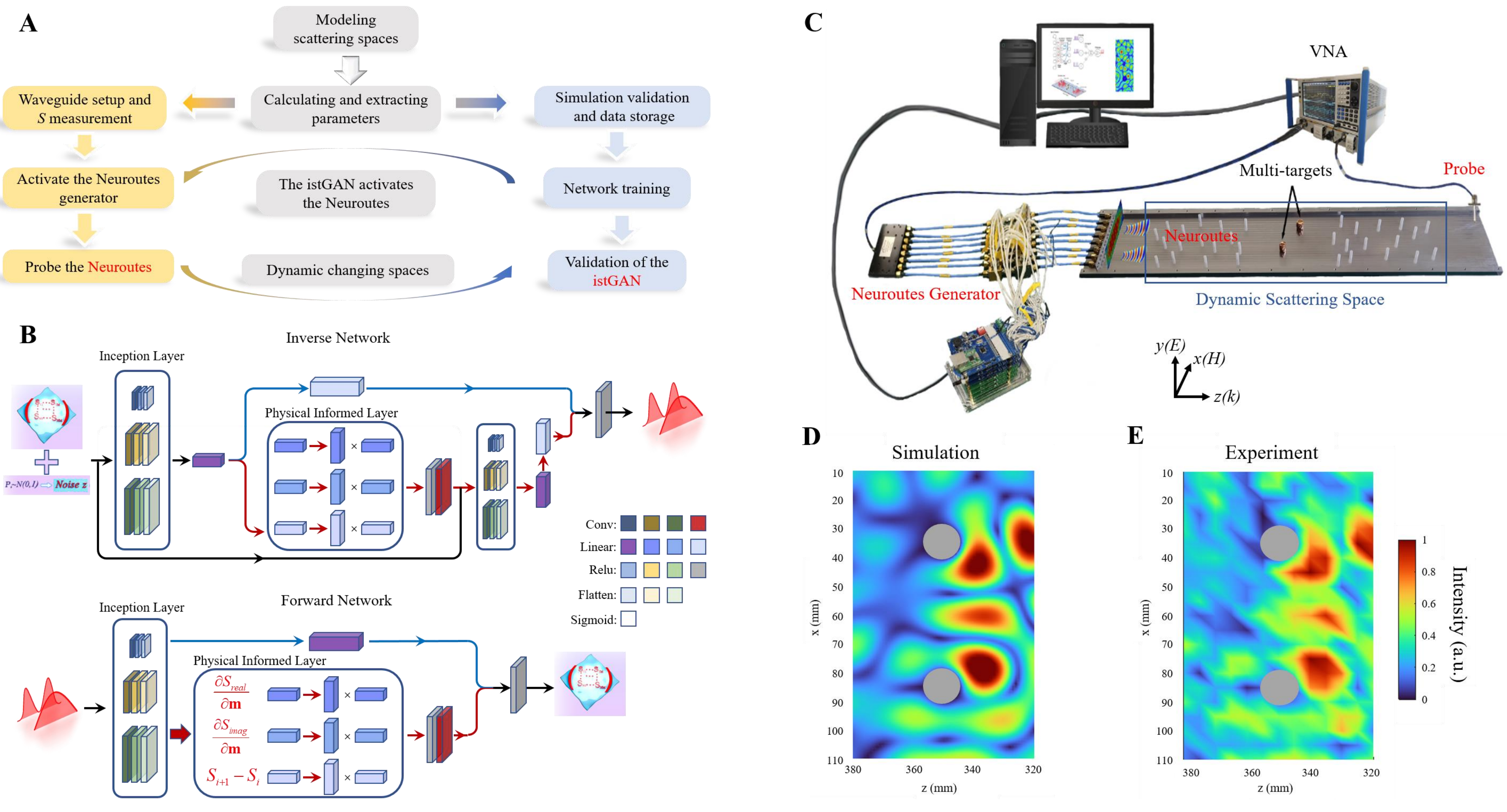
A
Modeling scattering spaces
Waveguide setup and S measurement
Calculating and extracting parameters
Simulation validation and data storage
Activate the Neuroutes generator
The istGAN activates the Neuroutes
Network training
Probe the Neuroutes
Dynamic changing spaces
Validation of the istGAN
B
Inverse Network
Inception Layer
Physical Informed Layer
Forward Network
Conv:
Linear:
Relu:
Flatten:
Sigmoid:
Noise z
C
VNA
Multi-targets
Probe
Neuroutes
Neuroutes Generator
Dynamic Scattering Space
y(E)
x(H)
z(k)
D
Simulation
E
Experiment
x (mm)
z (mm)
Intensity (a.u.)

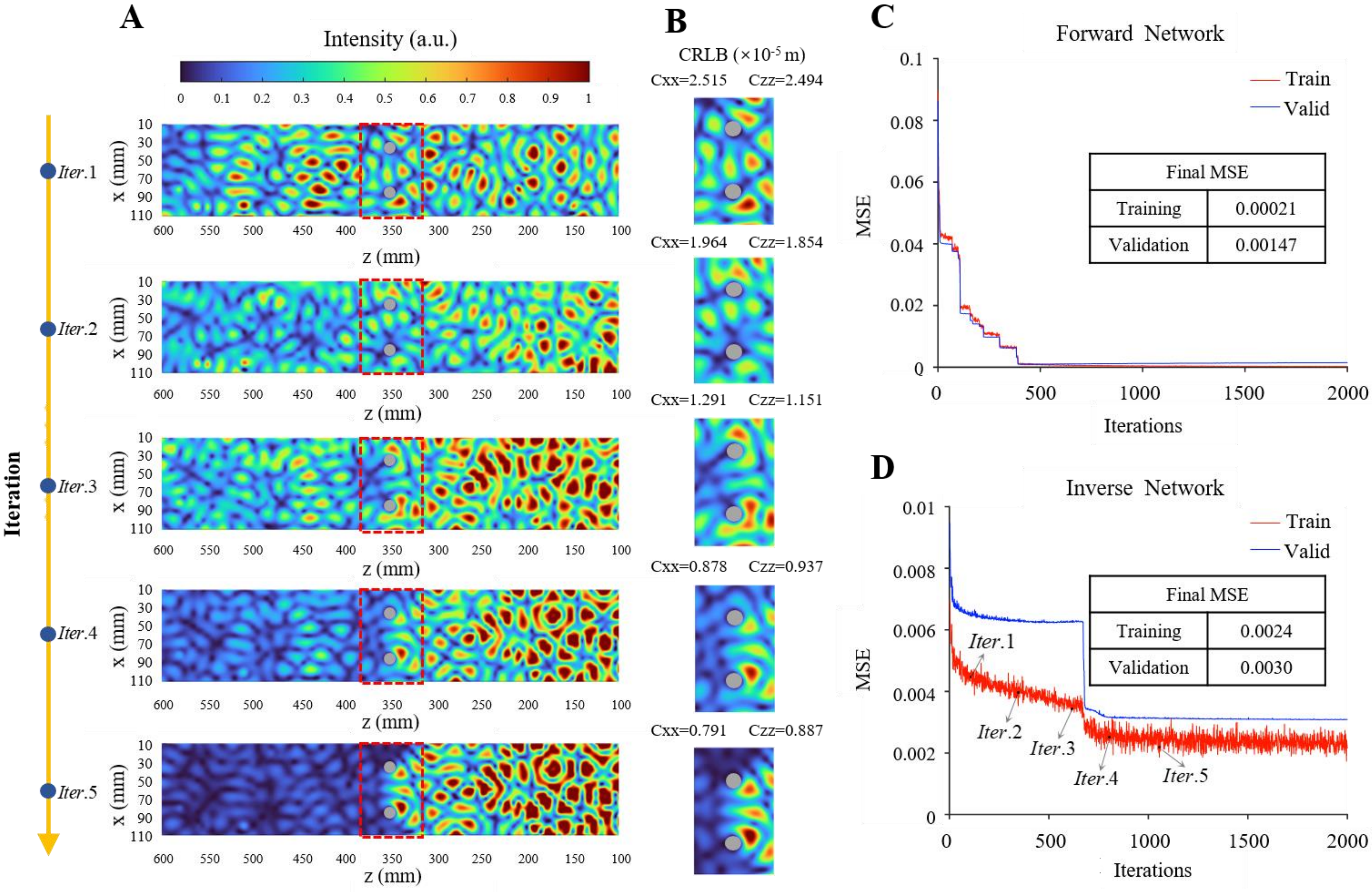

A
Intensity (a.u.)
0 0.1 0.2 0.3 0.4 0.5 0.6 0.7 0.8 0.9 1
Iteration
Iter.1
Iter.2
Iter.3
Iter.4
Iter.5
x (mm)
10 30 50 70 90 110
600 550 500 450 400 350 300 250 200 150 100
z (mm)
B
CRLB (×10⁻⁵ m)
Cxx=2.515 Czz=2.494
Cxx=1.964 Czz=1.854
Cxx=1.291 Czz=1.151
Cxx=0.878 Czz=0.937
Cxx=0.791 Czz=0.887
C
Forward Network
Train
Valid
MSE
Iterations
Final MSE
Training 0.00021
Validation 0.00147
D
Inverse Network
Train
Valid
MSE
Iterations
Final MSE
Training 0.0024
Validation 0.0030

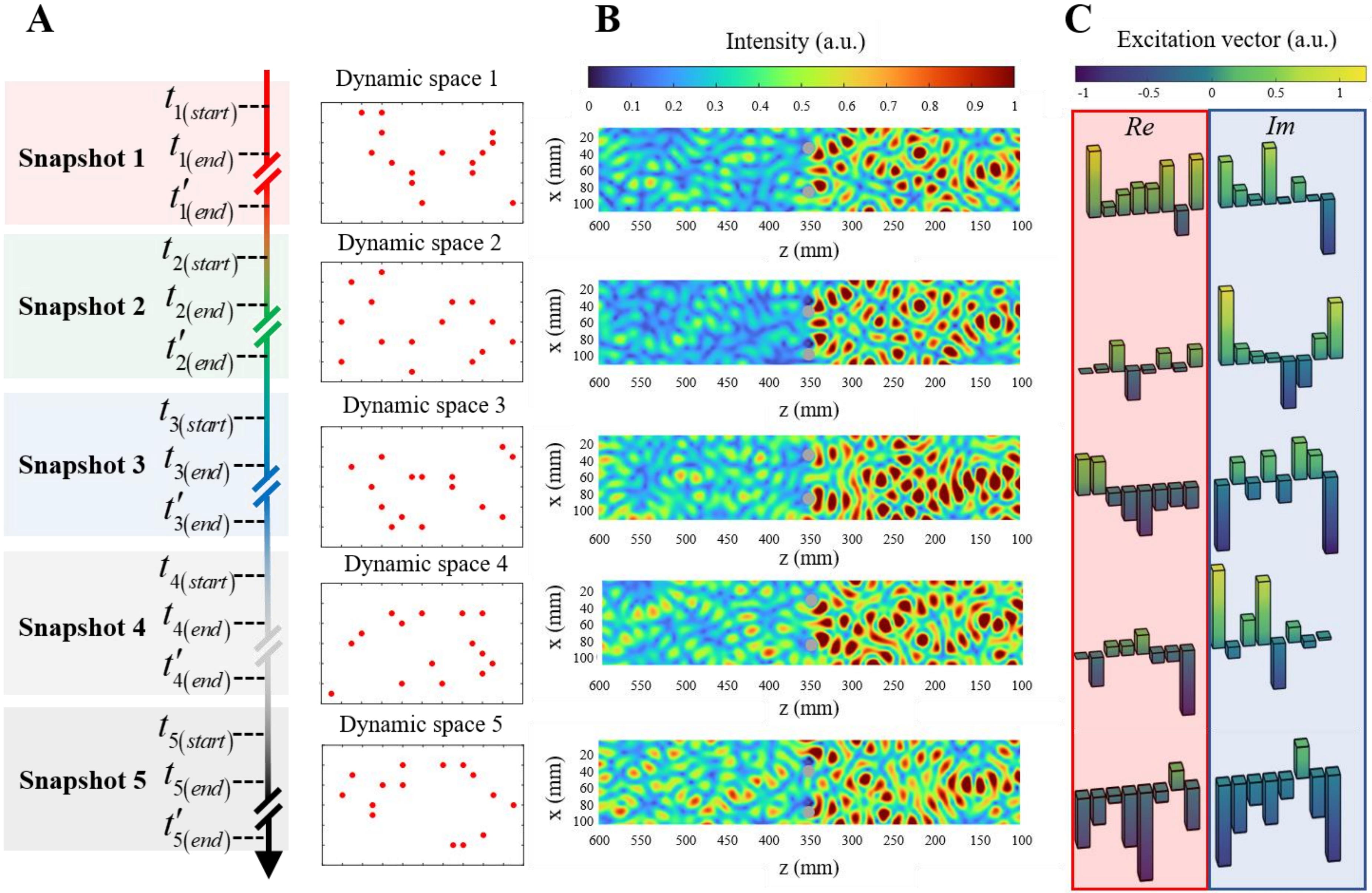

A
Snapshot 1
Snapshot 2
Snapshot 3
Snapshot 4
Snapshot 5
Dynamic space 1
Dynamic space 2
Dynamic space 3
Dynamic space 4
Dynamic space 5
B
Intensity (a.u.)
x (mm)
z (mm)
C
Excitation vector (a.u.)
Re
Im

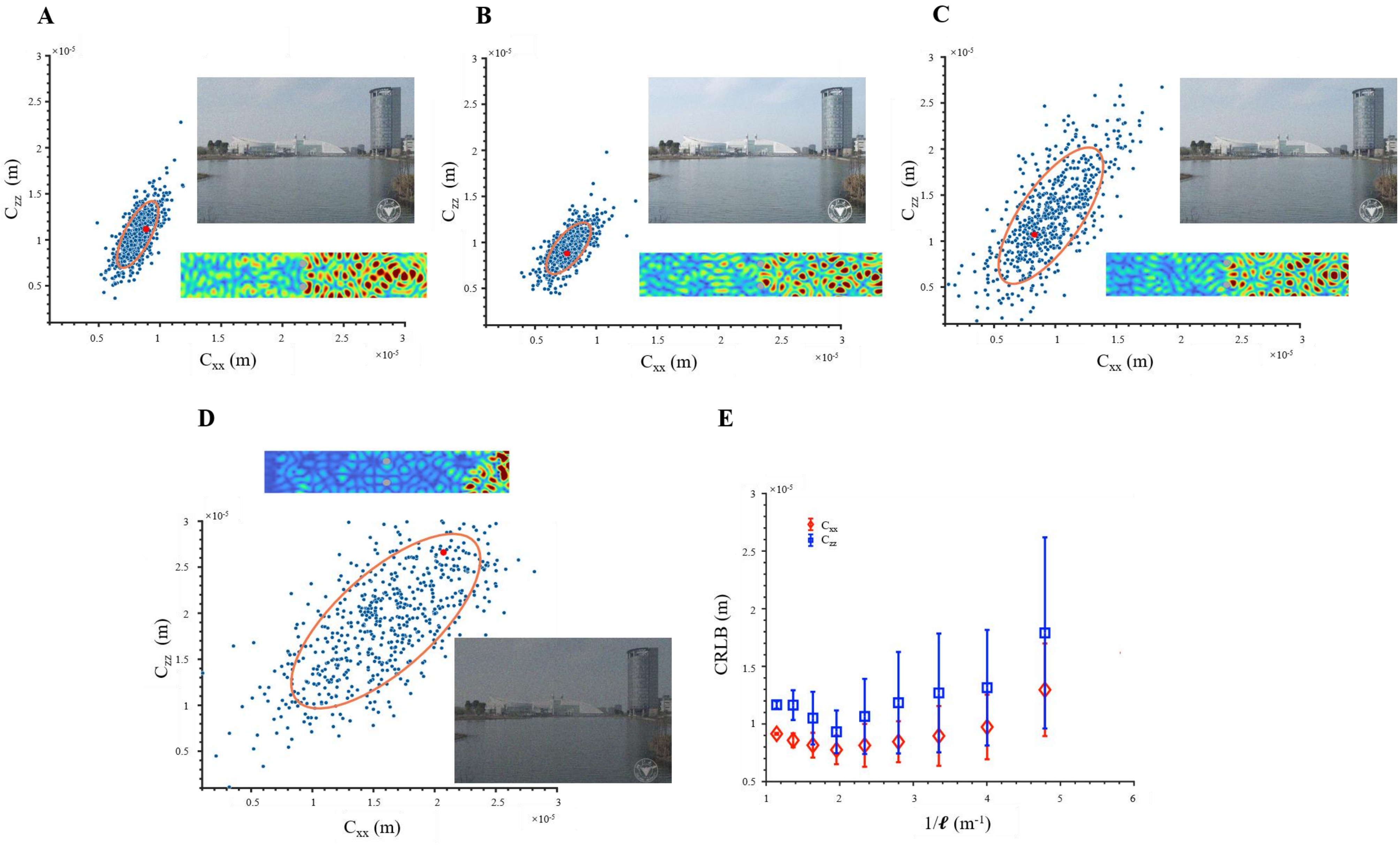
A
B
C
D
E
×10^-5
C_zz (m)
C_xx (m)
CRLB (m)
C_xx
C_zz
1/ℓ (m^-1)

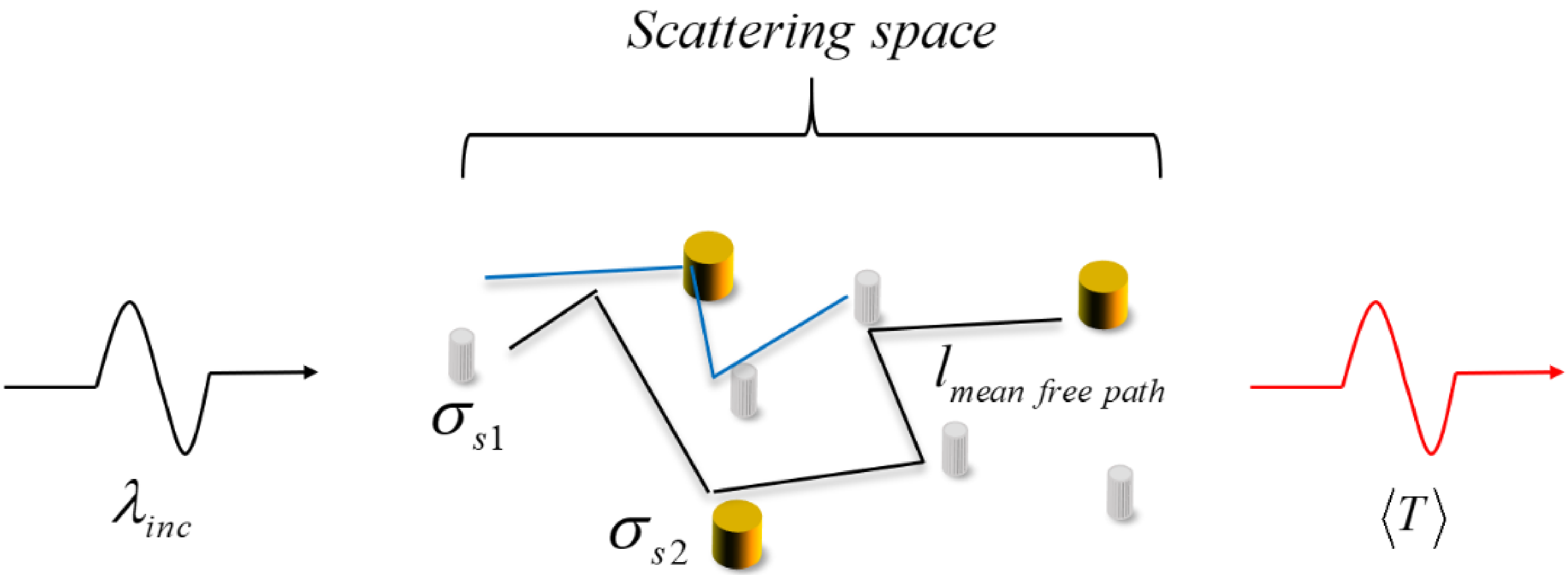

Scattering space
λ_{inc}
σ_{s1}
σ_{s2}
$l_{mean\ free\ path}$
$\langle T \rangle$

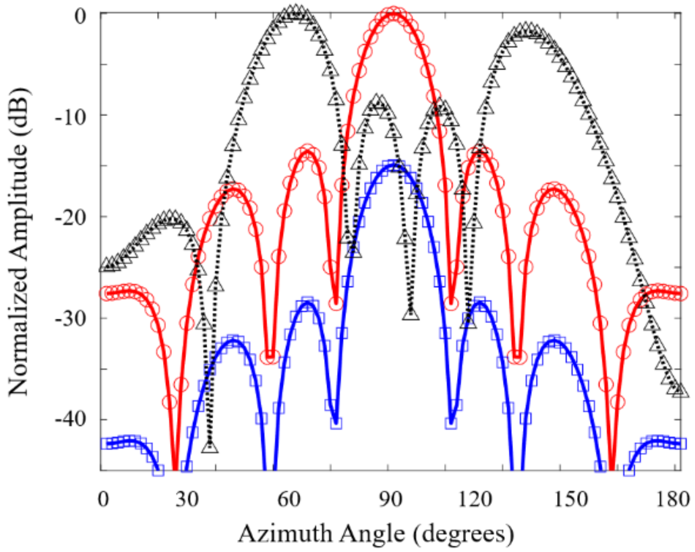

0
-10
-20
-30
-40
Normalized Amplitude (dB)
0
30
60
90
120
150
180
Azimuth Angle (degrees)

(a)

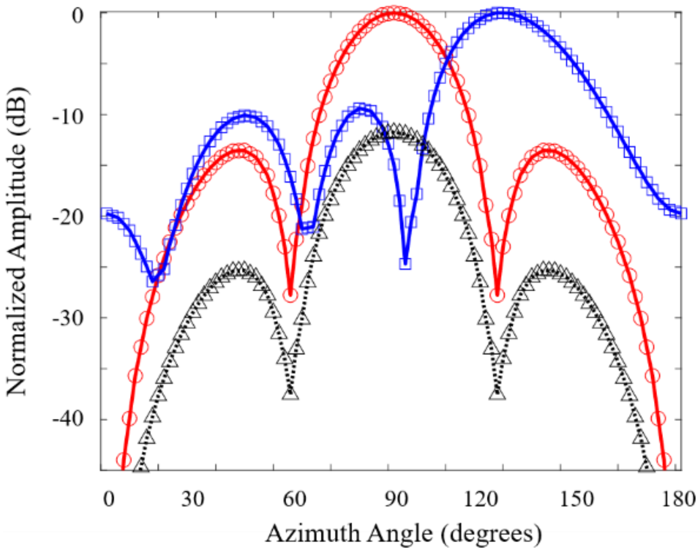

0
-10
-20
-30
-40
Normalized Amplitude (dB)
0
30
60
90
120
150
180
Azimuth Angle (degrees)

(b)

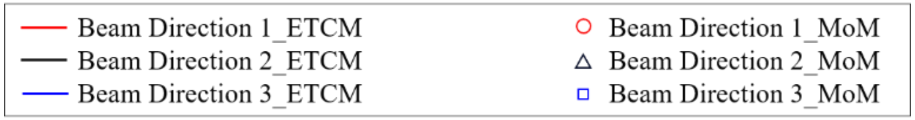

Beam Direction 1_ETCM
Beam Direction 2_ETCM
Beam Direction 3_ETCM
Beam Direction 1_MoM
Beam Direction 2_MoM
Beam Direction 3_MoM

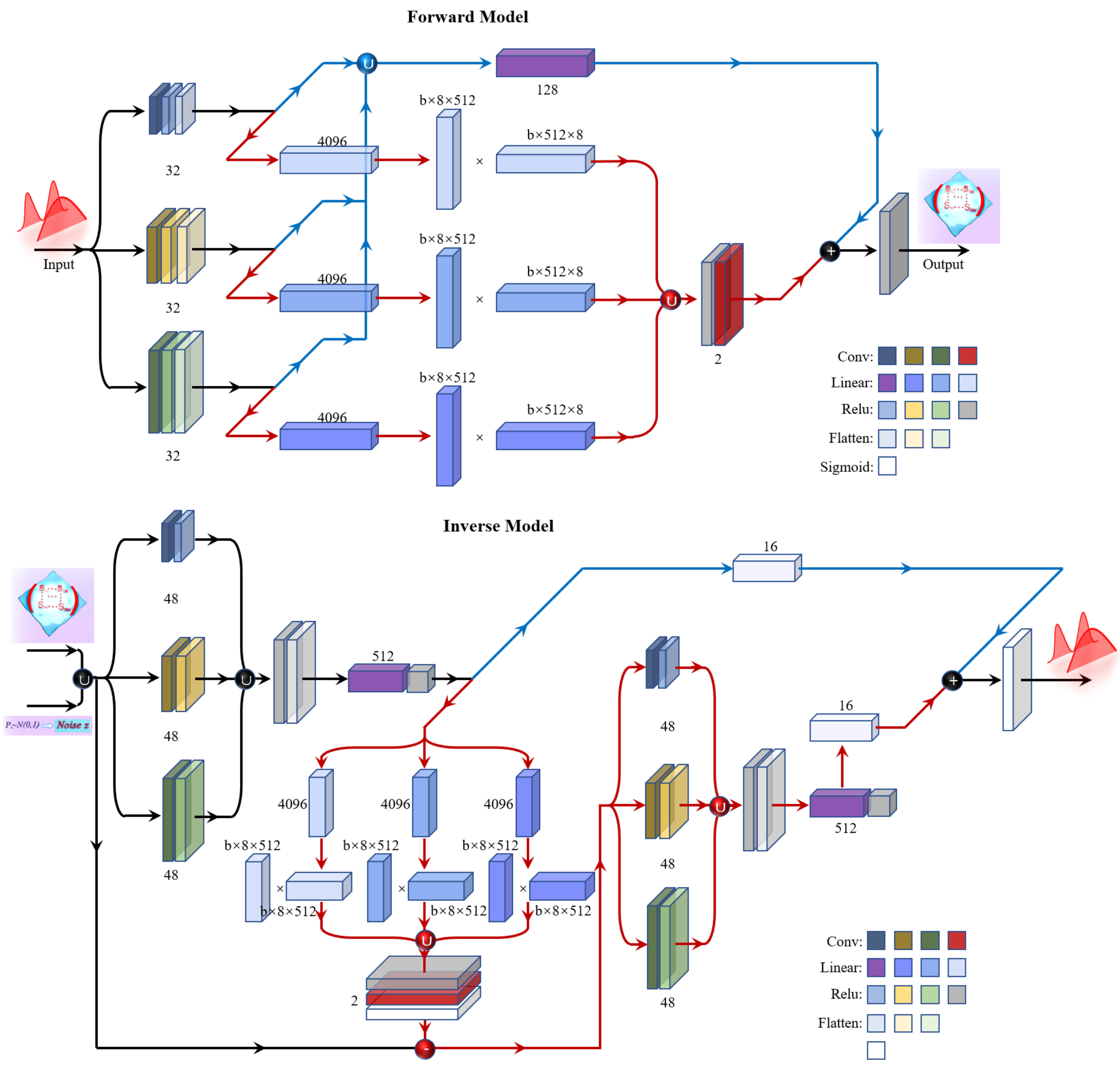

Forward Model
Input
32
32
32
4096
4096
4096
b×8×512
b×8×512
b×8×512
b×512×8
b×512×8
b×512×8
128
2
Output
Conv:
Linear:
Relu:
Flatten:
Sigmoid:
Inverse Model
48
48
48
512
16
4096
4096
4096
b×8×512
2
48
48
48
512
16
Noise z
Conv:
Linear:
Relu:
Flatten:

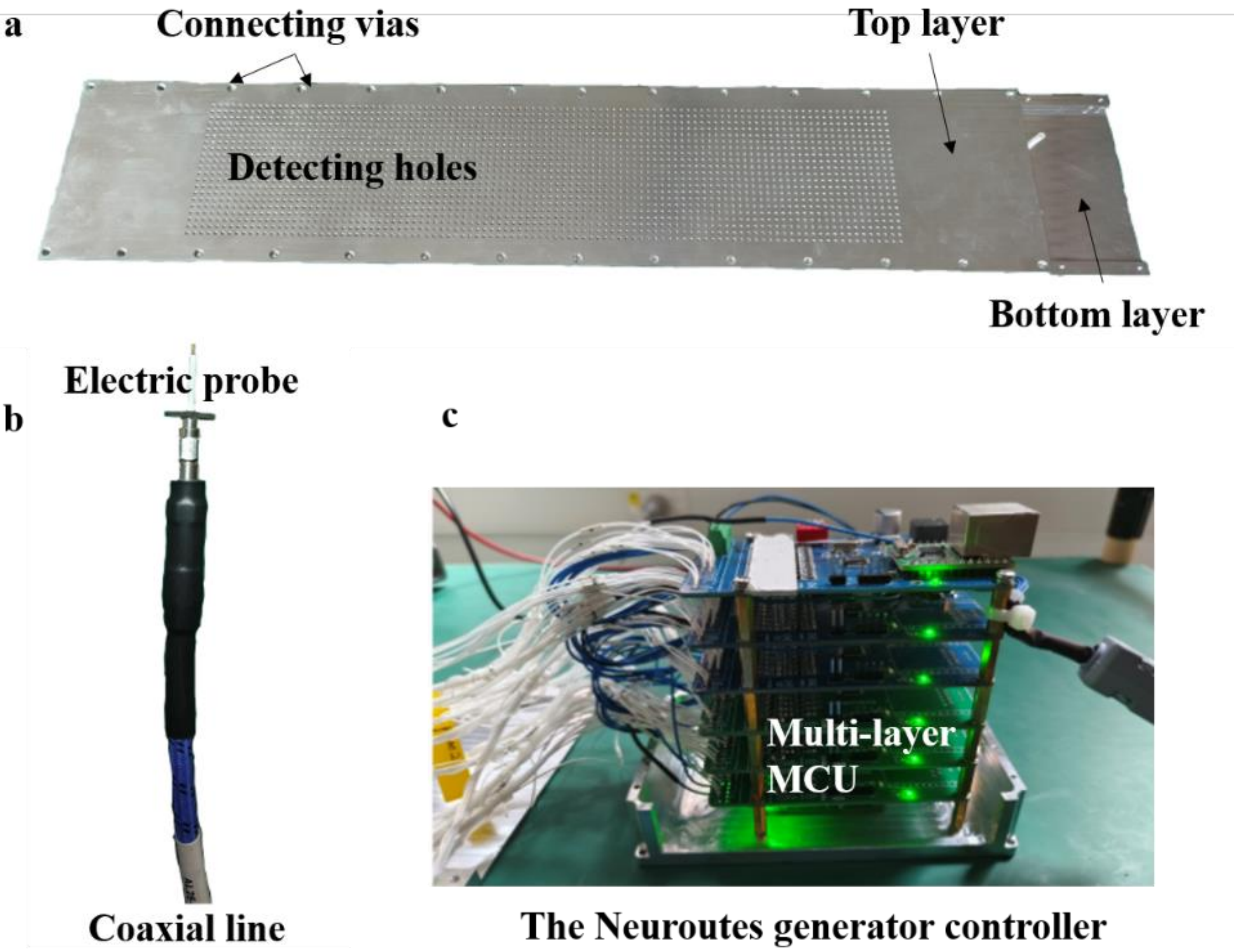
a
Connecting vias
Top layer
Detecting holes
Bottom layer
Electric probe
b
c
Multi-layer
MCU
Coaxial line
The Neuroutes generator controller

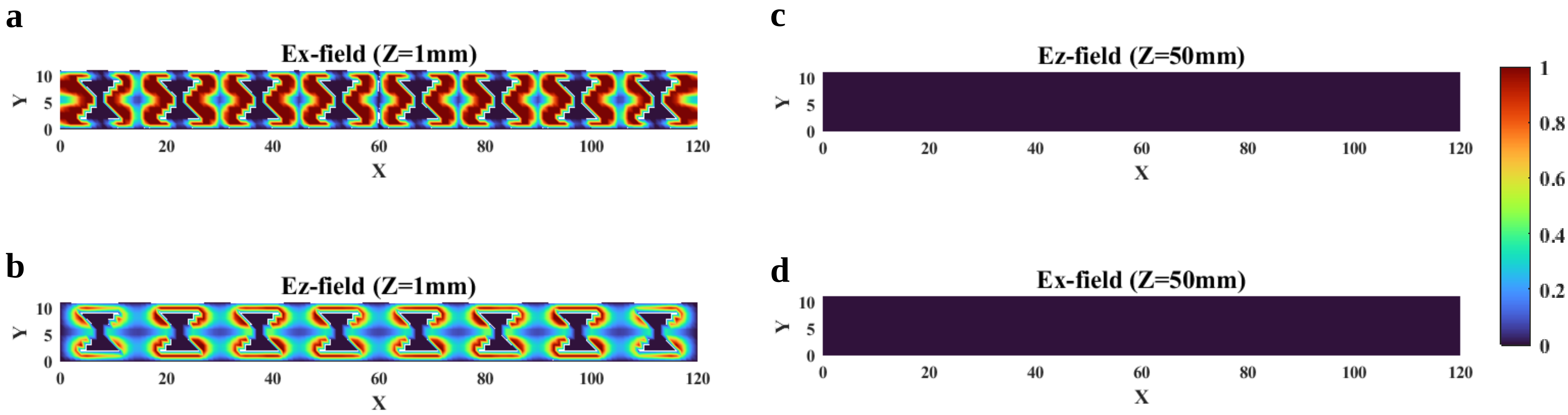

a
Ex-field (Z=1mm)
b
Ez-field (Z=1mm)
c
Ez-field (Z=50mm)
d
Ex-field (Z=50mm)
Y
X
0
5
10
20
40
60
80
100
120
1
0.8
0.6
0.4
0.2

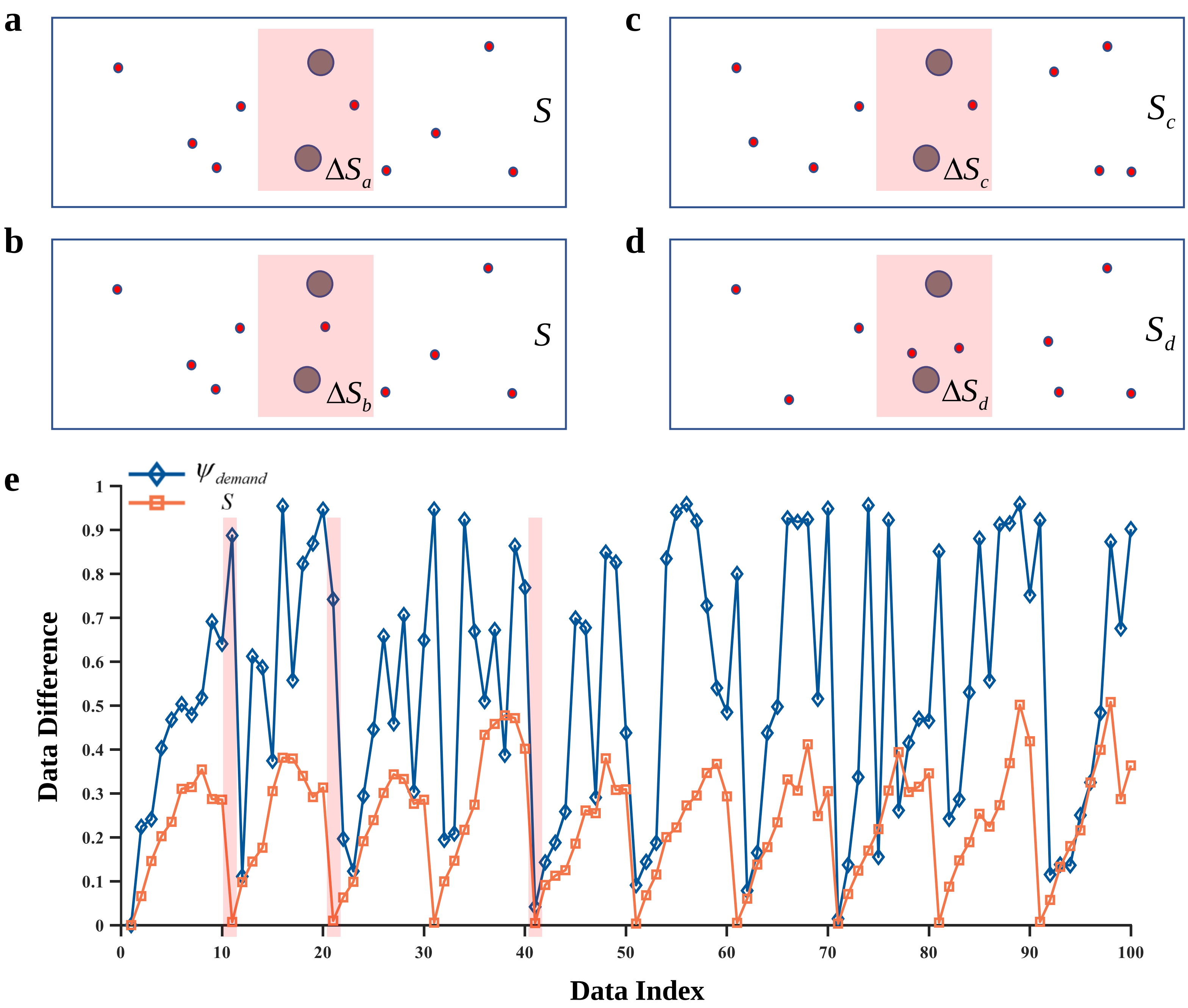

a
b
c
d
e
S
ΔS_a
ΔS_b
S_c
ΔS_c
S_d
ΔS_d
ψ_{demand}
Data Difference
Data Index

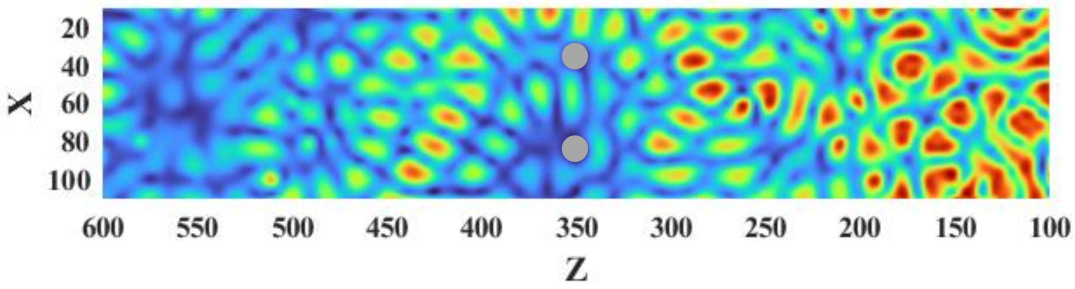
20
40
60
80
100
X
600
550
500
450
400
350
300
250
200
150
100
Z

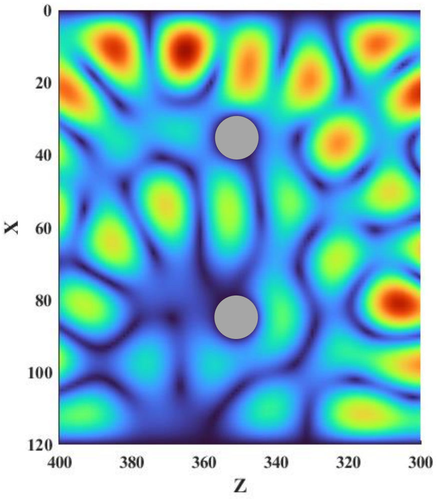
0
20
40
60
80
100
120
X
400
380
360
340
320
300
Z

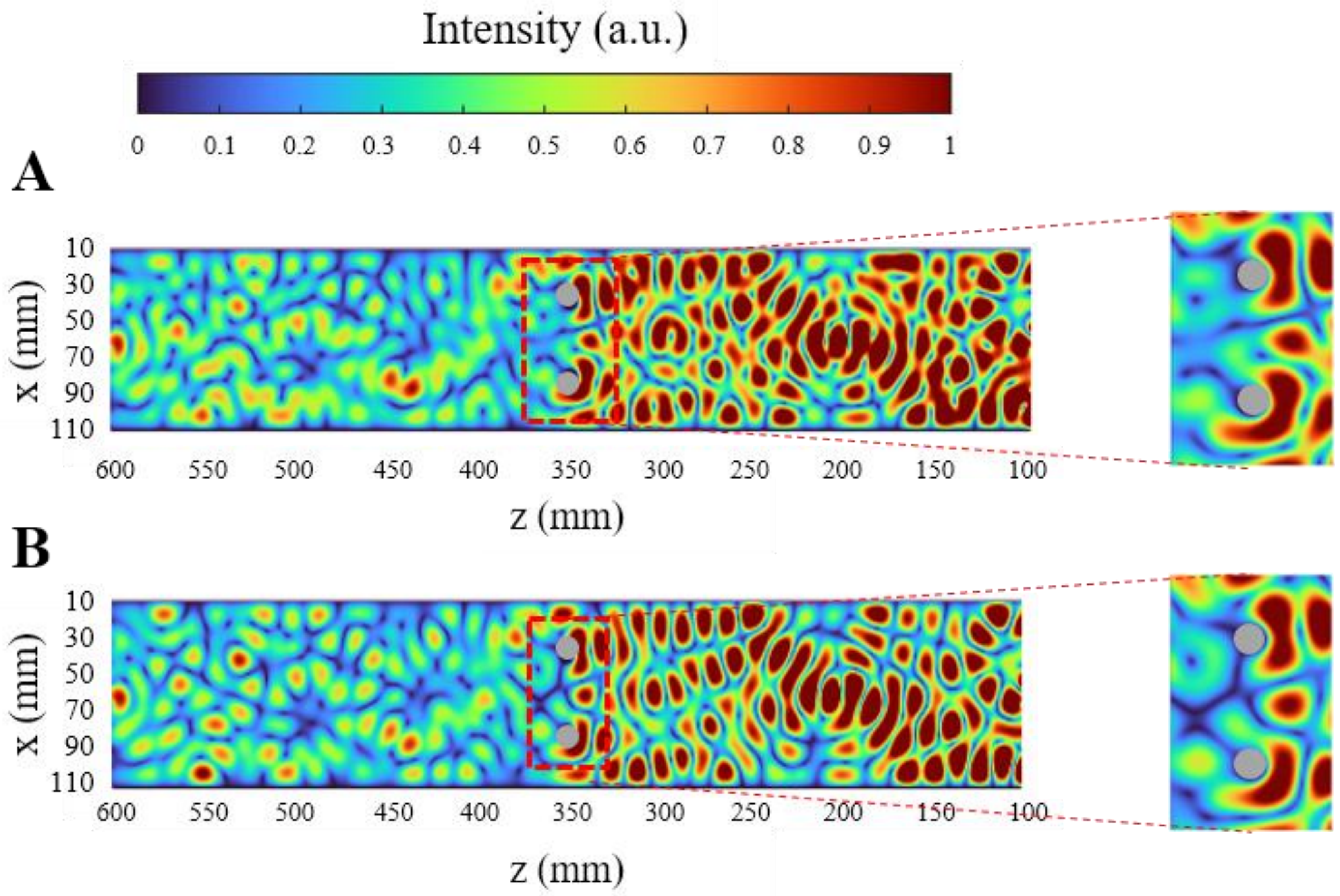

Intensity (a.u.)
0
0.1
0.2
0.3
0.4
0.5
0.6
0.7
0.8
0.9
1
A
x (mm)
10
30
50
70
90
110
600
550
500
450
400
350
300
250
200
150
100
z (mm)
B
x (mm)
10
30
50
70
90
110
600
550
500
450
400
350
300
250
200
150
100
z (mm)

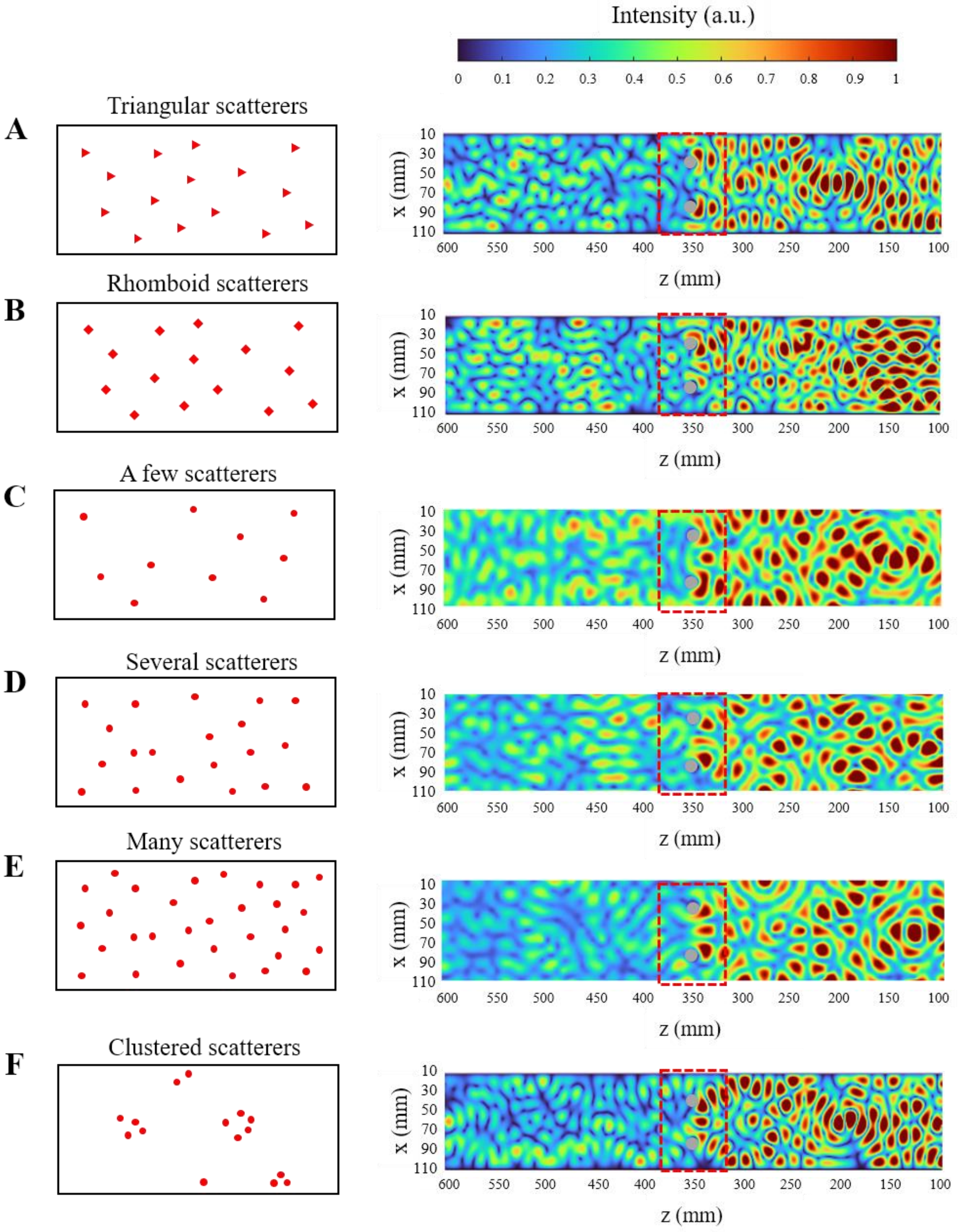
Intensity (a.u.)
0
0.1
0.2
0.3
0.4
0.5
0.6
0.7
0.8
0.9
1
A
Triangular scatterers
B
Rhomboid scatterers
C
A few scatterers
D
Several scatterers
E
Many scatterers
F
Clustered scatterers
x (mm)
10
30
50
70
90
110
600
550
500
450
400
350
300
250
200
150
100
z (mm)

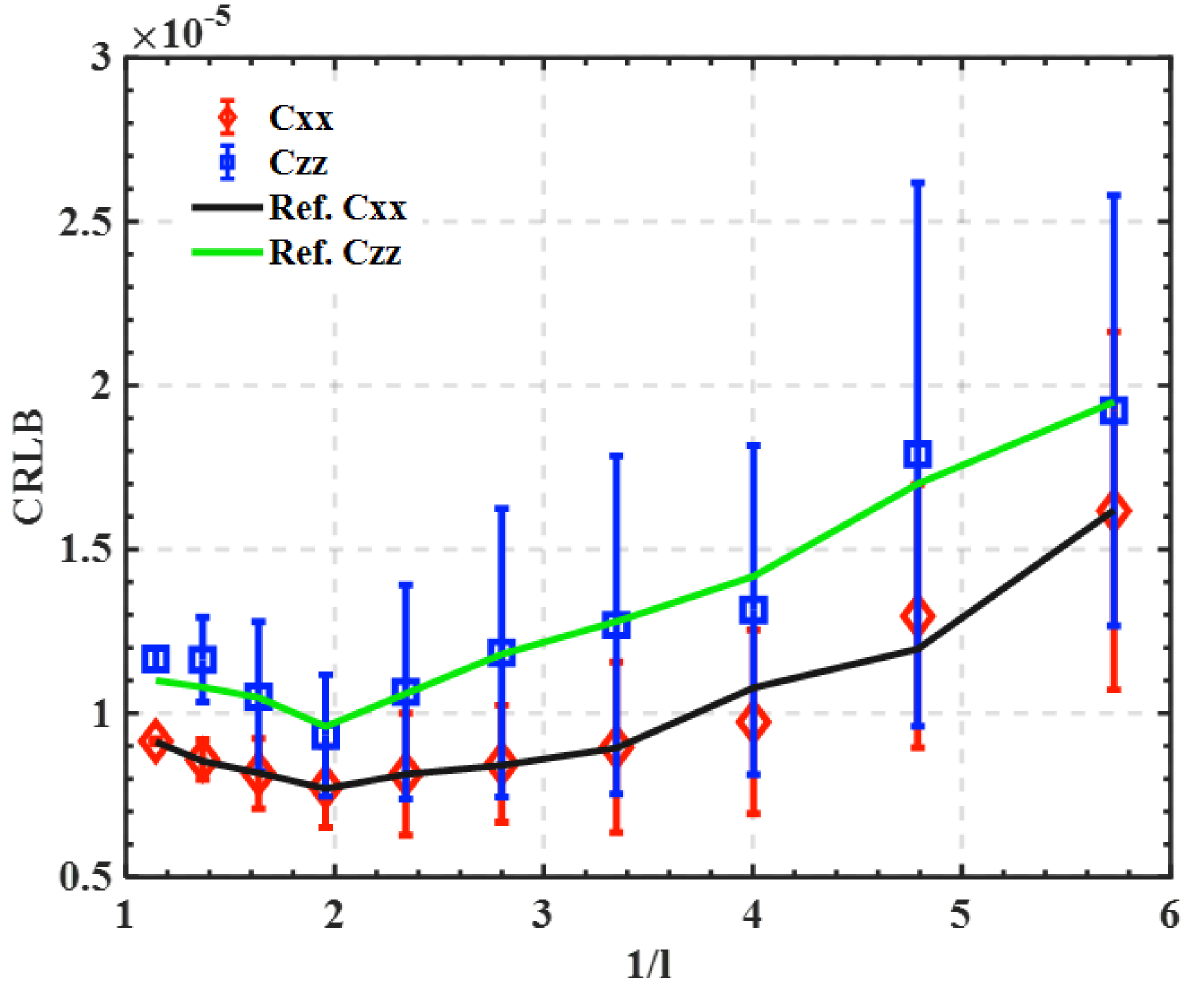

×10^-5
3
2.5
2
1.5
1
0.5
CRLB
1
2
3
4
5
6
1/l
Cxx
Czz
Ref. Cxx
Ref. Czz
